# Multi-omics data integration from patients with carotid stenosis illuminates key molecular signatures of atherosclerotic instability

**DOI:** 10.1186/s13073-026-01601-5

**Published:** 2026-02-06

**Authors:** Vivek Das, Sampath Narayanan, Xiang Zhang, Otto Bergman, Djordje Djordjevic, Malin Kronqvist, Melody Chemaly, Glykeria Karadimou, Sofija Sundman, Inika Prasad, Andrew J. Buckler, Karin Conde Knape, Natasha Barascuk Michaelsen, Ulf Hedin, Ljubica Matic

**Affiliations:** 1 Novo Nordisk A/S Måløv, Måløv, Denmark; 2 Department of Molecular Medicine and Surgery, Vascular Surgery, BioClinicum J8:20, Karolinska Institutet, Solna, Stockholm SE-171 76 Sweden; 3 MedTechLabs, BioClinicum, Karolinska University Hospital, Solna, Sweden

**Keywords:** Multi-omics integration, Atherosclerosis, Plaques, Biomarkers, Therapy

## Abstract

**Background:**

Understanding the pathophysiology of unstable atherosclerosis is imperative to prevent myocardial infarction and stroke. Here, we used multi-omics integration to identify key molecular targets with diagnostic and therapeutic potential.

**Methods:**

Biobank of Karolinska Endarterectomies encompassing patients with symptomatic (S) and asymptomatic (AS) carotid atherosclerosis was the main resource. Plaques, peripheral blood monocytes and plasma sampled locally from around plaque or periphery of *n* > 700 individuals, were profiled by transcriptomics, proteomics and metabolomics. A supervised machine learning method DIABLO was used for patient data integration. Multi-omics layers were integrated separately across local and peripheral disease sites, and their intersection, with stratification for symptomatology. Identified analytes were investigated using scRNAseq, clinical and outcome data.

**Results:**

In peripheral circulation, FABP4, IL6, Bilirubin and Sphingomyelin were the most prominent analytes. F11, ANGPTL3, ICOSLG, ITGB1 and Sphingomyelin were enriched in the local disease site, while FABP4, C1R, IL6, Bilirubin and Sphingomyelin appeared at the intersection. Coagulation, necroptosis, inflammation and cholesterol metabolism were confirmed as key pathways determining symptomatology. Clinical analyses showed an impact of lipid-lowering therapy on ICOSLG expression, anti-hypertensives on plasma FABP4 and BLVRB levels, anti-diabetics on plasma Sphingomyelins, while no medications affected ANGPTL3. Association with future adverse events was shown for plasma Bilirubin, Sphingomyelin, ANGPTL3 and ICOSLG plaque levels. Open-source target analyses suggested genetic involvement of F11, C1S, EGFR, IL6, ANGPTL3 in the disease.

**Conclusions:**

Using an innovative, multi-modal data integration machine learning framework, this study provides confirmatory and novel information on mechanisms behind atherosclerotic instability. The findings raise possibilities for translational prioritizations to aid personalized medicine.

**Supplementary Information:**

The online version contains supplementary material available at 10.1186/s13073-026-01601-5.

## Background

Despite the profound impact of atherosclerotic cardiovascular disease (ASCVD) on global mortality and morbidity from myocardial infarction (MI) or ischemic stroke (IS), drug development in this area has plateaued in recent years. Based on the Framingham Heart Study [1], ASCVD research has for decades focused on pathophysiological pathways related to various risk factors, resulting in the development of successful preventive strategies targeting lipids, thrombosis, diabetes, hypertension and lately also inflammation. Nevertheless, there is a considerable residual risk with 5–10% optimally-managed patients experiencing a major cardio- or cerebro-vascular event (MACCE) three years after the initial event and a life-time risk of up to 50–60% [2]. More specifically, asymptomatic (AS, stable) carotid disease is associated with an annual stroke risk of 1–3%, whereas symptomatic (S, unstable) patients with either transient ischemic attack (TIA) or minor stroke (MS) have a more than 10-fold increased risk [1]. While current prevention strategies have clearly curbed mortality rates, substantial risk remains and importantly, existing treatments lack specificity for diverse patient subgroups. Thus, there is a need to subcategorize patients, where precise, individualized therapeutic strategies grounded in vascular disease biology can be applied.

Atherogenesis is critically associated with hyperlipidaemia, infiltration and retention of ApoB containing lipoproteins in the artery wall, triggering inflammatory responses with endothelial dysfunction and infiltration of monocytes, which develop into foam cells and form fatty streaks [3–5]. Cytokines and chemokines secreted by macrophages over time promote recruitment of other inflammatory cells [5], which gradually leads also to the activation of medial smooth muscle cells (SMCs) into a secretory and replicating phenotype that engage in intimal remodelling and fibrous cap formation [6, 7]. Progressive inflammation contributes to plaque instability by inhibiting collagen production, stimulating neovessel formation and intraplaque hemorrhage, as well as matrix metalloproteinases that degrade the fibrous cap, which altogether may lead to its rupture, atherothrombosis and fatal clinical manifestations [8, 9].

In previous years, analyses of human atherosclerosis from single-omics, i.e. transcriptomic or proteomic datasets, combined with both patient and plaque phenotyping, have facilitated discoveries of novel molecular targets and pathways in plaque instability [10, 11]. More recently, advanced large-scale studies have mapped genetic risk loci across diverse populations [12–14], performed in-depth molecular subtyping [13, 15] and dissected cell-specific disease mechanisms [6, 16–23], resulting in identification of several subclinical phenotypes of ASCVD [24]. Especially single-cell RNA sequencing (scRNAseq) has improved the characterization of different cell populations in atherosclerosis, with identification of various T-cells, macrophages and SMCs subsets. Moreover, single-cell atlases of ASCVD have revealed that, in addition to atherogenic dyslipidemia and systemic inflammation, a considerable portion of genetic risk can be explained by the dysfunction of endothelial cells and SMCs [17, 25–27], raising a notion that cells of the vessel wall could be a source of novel biomarkers or therapeutic targets.

However, few holistic systems biology approaches have been applied in the field so far, although efforts to elucidate complex disease-underlying mechanisms by integrating different layers of omics information using machine learning technologies have shown promise in other areas [28]. To this end, DIABLO (Data Integration Analysis for Biomarker discovery using Latent cOmponents) has been utilized, as a novel integrative method that seeks for common information across different high-dimensional data types. DIABLO is based on pairwise linear combinations of factors that reduces dimensionality of the data, captures biological variability and identifies correlated molecular analytes associated with categorical phenotypes. It has been shown that DIABLO identifies features with superior biological relevance, achieving predictive performance that supersedes other state-of-the-art approaches [29].

**Fig. 1:**
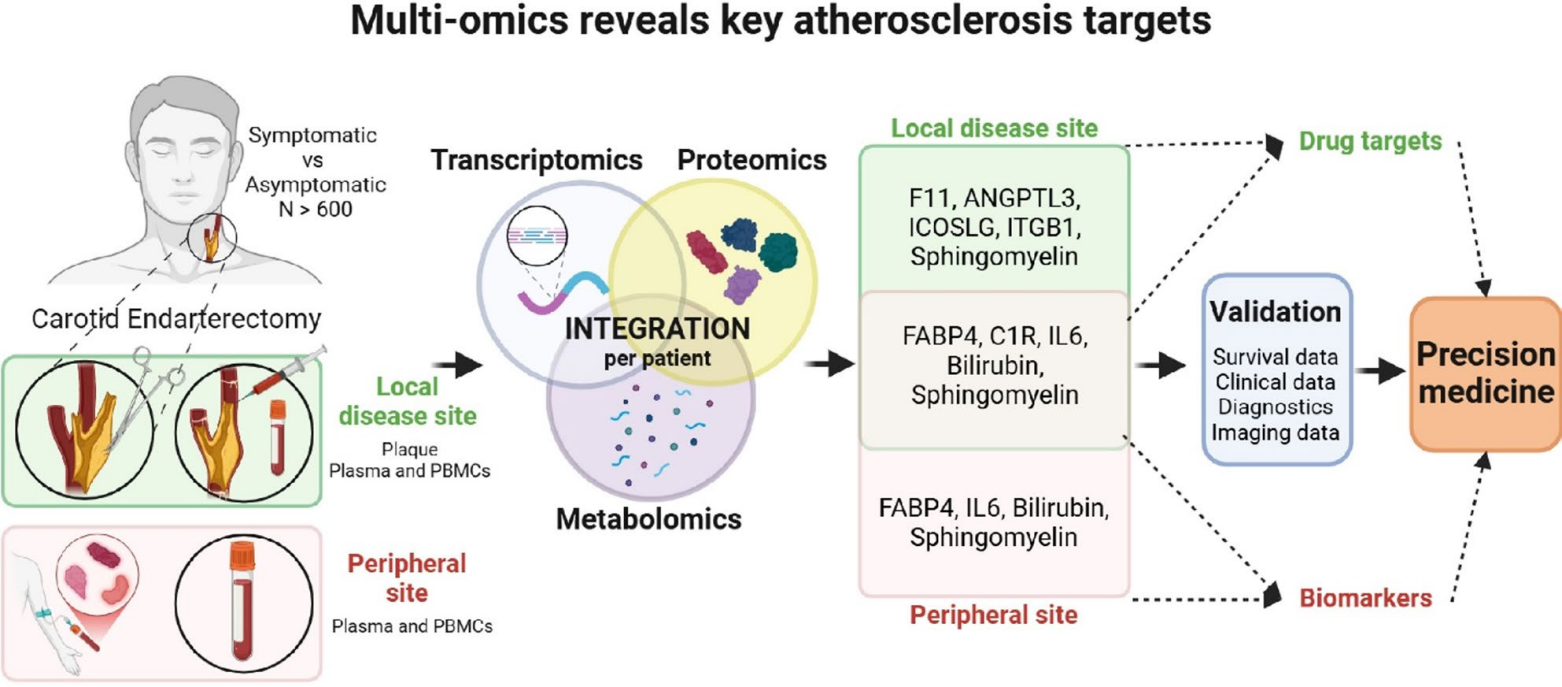
Visual summary of the study. We performed first-of-a-kind, orthogonal, per-patient, multi-omics integration from a large carotid stenosis biobank, with an aim to identify key molecular signatures and pathways of human atherosclerotic instability. The complex multi-omics design coupled with deep-data analyses, enabled the discovery of numerous confirmatory and novel molecular signatures implicated in patient symptomatology. Extended association analyses elucidated their cellular sources, associations with plaque morphology, clinical biochemistry, medication and long-term patient outcomes. The findings are interesting for further investigation with respect to druggable targeting or plasma biomarkers, altogether leading to improved patient phenotyping and precision medicine potential in cardiovascular disease

In this study, our objective was to accelerate the identification of novel biomarker and therapeutic candidates specifically targeting instability in late-stage atherosclerosis. To this end, we aimed to leverage the power of multi-omics integrations from a large human biobank using DIABLO, never previously attempted on this scale and complexity in the field. Novel bioinformatic workflows for multi-dimensional disease assessment and target identification were developed by combining global transcriptomic, proteomic and metabolomic profiling of matched plaques, PBMCs and plasma from patients undergoing stroke-preventive carotid endarterectomy (CEA), utilizing the Biobank of Karolinska Endarterectomies (BiKE) [30]. The synergy of orthogonal omics datasets analyzed here was shown to be necessary for unravelling the deep mechanistic insights that underly heterogeneity in S vs. AS patients, as well as locally around the lesion vs. in peripheral circulation. The generated results illuminate both classical and novel aspects of ASCVD and enable a more comprehensive understanding of this patient population (Fig. 1).

## Methods

### Human carotid plaque cohort (Biobank of Karolinska Endarterectomies)

Patients undergoing carotid endarterectomy (CEA) at the Departments of Vascular Surgery from both Karolinska University Hospital or Södersjukhuset (affiliated to BiKE in 2008), Stockholm, Sweden were consecutively included in the Biobank of Karolinska Endarterectomies (BiKE) and clinical data recorded upon admission (demographic data presented in Table S1). Symptomatic (S) patients were categorized as those having a minor stroke (MS), transient ischemic attack (TIA), or ipsilateral ocular symptoms (*amaurosis fugax*, AFX), while patients without qualifying symptoms within 6 months prior to surgery were considered asymptomatic (AS). Plaques and blood were collected during surgery and retained within BiKE. “Local” samples were retrieved by aspirating blood from the carotid artery lumen adjacent to the plaque 5 min after artery cross clamping and before arteriotomy, as previously described [12]. Plaques were divided transversally at the most stenotic part and the proximal half of the plaque was frozen at -80 °C immediately after surgery and later used for transcriptomic or proteomic profiling. The distal half was fixed in 4% zinc-formaldehyde and processed for histology. Blood was processed according to standard procedures for separation of monocyte cell fraction and RNA isolation. Briefly, peripheral blood mononuclear cells (PBMCs) were isolated from blood collected at surgery *via* density gradient centrifugation through Ficoll-Paque (Vacutainer CPT, Becton-Dickinson, Franklin Lakes, NJ). PBMCs were processed using RLT buffer (Qiagen, Valencia, CA) before freezing at -80 °C.

In this study, transcriptomic dataset analyses were performed in a total of *n* = 423 plaques (343 S + 80 AS patients), *n* = 165 local PBMCs (136 S + 29 AS patients) and *n* = 399 peripheral PBMCs (328 S + 71 AS patients). Proteomic analyses were done in a total of *n* = 598 peripheral (479 S + 119 AS patients) and *n* = 199 local plasma samples (166 S + 33 AS patients). Metabolomic analyses were performed in a total of *n* = 640 peripheral (510 S + 130 AS patients) and *n* = 156 local plasma samples (134 S + 22 AS patients).

BiKE is today one of the largest tissue cohorts for carotid disease and has been extensively described in numerous prior publications [30].

### Material quality assurance for omics analyses

All biomaterial used for omics analyses was analysed at the same time point, regardless of how long it was stored, and it was strictly quality controlled as per standard quidelines. Any samples that did not show satisfactory quality were never sent for the omics profiling. Likewise, any samples that did not pass the post-processing data quality controls, were excluded from bioinformatic analyses. Importantly, all proteomic and metabolomic profiling analyses were done in one single batch, while transcriptomic profiling was done in several batches and batch corrections were performed prior to any single- or multi-omics analyses. There was no imputation of missing values in any of the omics or other datasets in this study.

### BiKE clinical, epidemiological and follow-up patient data collection

Individual data for death, major adverse cardio- and cerebro-vascular events (MACCEs), comorbidities and blood parameters were extracted from the national registries (i.e. Swedish Cause of Death Register, Swedish Hospital Discharge Register, etc.), clinical patient charts and BiKE database, and stored using a system of individual pseudonymization for compliance with the General Data Protection Regulations (GDPR). Individual data such as diagnostic codes from the International Classification of Diseases (ICD-10), were extracted for every hospitalization and visit to the specialist outpatient clinic from the National Patient Register. The following data were extracted from the clinical database of BiKE and medical records: patient’s smoking habits, symptomatology prior to surgery, body mass index, hypertension, kidney function and medications. The start of inclusion was 31st August 1998, and the end was 12th December 2017. Data were extracted from national registries on 22nd January 2018. Follow-up was calculated from the date of the patient’s first carotid surgery. Patients treated with CEA bilaterally were included at the first procedure.

### Transcriptomic analyses - *RNA sequencing*

RNA was prepared using Qiazol Lysis Reagent (Qiagen, Hilden, Germany) and purified by RNeasy Mini kit (#74106, Qiagen, Germany), including DNase digestion. The concentration was measured using Nanodrop ND-1000 (Thermo Scientific, Waltham, MA) and quality estimated by a Bioanalyzer capillary electrophoresis system (Agilent Technologies, Santa Clara, CA). The library for bulk RNA sequencing of RNA from plaques and PBMCs was prepared using TruSeq stranded total RNA with RiboZero Globin treatment (# 20020612/20020613, Illumina Inc.). Paired-end 150 bp read length, NovaSeq 6000 system, S4 flow cells and v1 sequencing chemistry was used for sequencing at 20 mreads/sample. Libraries that yielded less data then aimed for were re-sequenced on SP flowcell. The Bcl to FastQ conversion was performed using bcl2fastq_v2.20.0.422 from the CASAVA software suite, followed by downstream analysis. The raw fastq files were processed using nf-core RNAseq pipeline. Human reference genome GRCh38 obtained from Ensembl was used for alignment. Gene level counts data of protein-coding and lincRNA genes were considered for all the downstream analysis. R package DESeq2 v1.34.0(51) was employed for differential expression analysis with adjustment for confounding factors such as age and gender performed using R/Bioconductor package DESeq2 v1.26.0. Pathway enrichment analysis was performed using the tool GSEA v4.1.0.

### Proteomic analyses - *plasma proteomics*

The following five Olink^®^ panels were used in plasma profiling: *Olink*^®^ *Target 96 Cardiometabolic*, *Olink*^®^ *Target 96 CVD II*, *Olink*^®^ *Target 96 CVD III*, *Olink*^®^ *Target 96 Development*, and *Olink*^®^ *Target 96 Immuno-Oncology*. This multiplex biomarker platform detected approximately 500 proteins in plasma. The platform uses proprietary Proximity Extension Assay (PEA) technology and a readout based on Next Generation Sequencing (NGS) in Illumina NovaSeq 6000. OlinkAnalyze package was used to perform the standard quality control (QC) on this panel of protein analytes using the NPX function and relevant QC parameters as suggested by the package vignette to remove the outlier samples that do not meet their standard quality flags. For multivariate analysis to identify differential proteins between S vs. AS groups, we used generalized linear mixed model package lme4. This analysis was run across 2 different sites, peripheral (EP) and local (STP). The final models were adjusted for sex + age. Only proteins with *p* value ≤ 0.05 were chosen for subsequent downstream analysis.

### Metabolomic analyses – *plasma metabolomics*

Plasma metabolomic analyses were performed at Metabolon, Morrisville, NC. Samples were prepared using the automated MicroLab STAR^®^ system from Hamilton Company. To remove protein, dissociate small molecules bound to protein or trapped in the precipitated protein matrix, and to recover chemically diverse metabolites, proteins were precipitated with methanol under vigorous shaking for 2 min (Glen Mills GenoGrinder 2000) followed by centrifugation. The resulting extract was divided into five fractions: two for analysis by two separate reverse phase (RP)/UPLC-MS/MS methods with positive ion mode electrospray ionization (ESI), one for analysis by RP/UPLC-MS/MS with negative ion mode ESI, one for analysis by HILIC/UPLC-MS/MS with negative ion mode ESI, and one sample was reserved for backup. Samples were placed briefly on a TurboVap^®^ (Zymark) to remove the organic solvent. Experimental samples were randomized across the platform run with QC samples spaced evenly among the injections. All methods utilized a Waters ACQUITY ultra-performance liquid chromatography (UPLC) and a Thermo Scientific Q-Exactive high resolution/accurate mass spectrometer interfaced with a heated electrospray ionization (HESI-II) source and Orbitrap mass analyzer operated at 35,000 mass resolution.

Raw data was extracted, peak-identified and QC processed using Metabolon’s hardware and software. Compounds were identified by comparison to library entries of purified standards or recurrent unknown entities. Metabolon maintains a library based on authenticated standards that contains the retention time/index (RI), mass to charge ratio (m/z), and chromatographic data (including MS/MS spectral data) on all molecules present in the library. Furthermore, biochemical identifications are based on three criteria: retention index within a narrow RI window of the proposed identification, accurate mass match to the library +/- 10 ppm, and the MS/MS forward and reverse scores between the experimental data and authentic standards. The MS/MS scores are based on a comparison of the ions present in the experimental spectrum to the ions present in the library spectrum.

The present dataset comprises a total of 1090 compounds of known identity (named biochemicals). Following log transformation, we again used lme4 as for proteomics, for multivariate analysis to identify differentially regulated metabolites between S vs. AS groups across EP and STP disease sites. The models were adjusted for sex and age. Only metabolites with *p* value ≤ 0.05 were chosen for subsequent downstream analysis with MetaboAnalyst 5.0 for functional enrichment.

### Multi-omics analyses - *general description*

DIABLO software from the mixOmics R package (version 6.25.1) was used for the entire analysis [29]. Normalized data across each omics type (transcriptomics, proteomics, metabolomics) were used as input for supervised DIABLO. The analysis was performed for 3 different combinations (peripheral circulation – Combination 1, local disease site – Combination 2, local plaque with peripheral circulation – Combination 3) to compare and identify cross-sectional molecular analytes that capture the difference between S vs. AS groups. Overall greater than 3000 features from across all omics types were used for analysis with DIABLO in each combination. Optimal number of supervised features across each omics type was determined by tune.block.spslda function. Greater than 28 000 models have been fitted for each component and each nrepeat, using “centroid.dist” parameter with a 10-fold cross validation to identify the optimal number of features across transcripts, proteins and metabolites used for downstream integration analysis. In DIABLO, each variable is assigned a weight (or loading) that indicates its contribution to the latent components. Higher weights signify greater importance in explaining the relationships between datasets. This contribution can also be interpreted as an effect size, and their direction indicates if this is towards one categorical phenotype vs. the other. For more clarity, DIABLO constructs latent components as linear combinations of variables from each dataset. The goal is to maximize the covariance between datasets, ensuring that the selected variables contribute to the shared structure.

A systematic assessment around the design matrix was performed that assumes the contribution from each omics layer for integration and feature selection between 0%, 10%, 20%, 30% and 40%. The final model was selected and fitted to block.splsda with a weighted design matrix of 30% contribution across omics types that can discriminate and explain the phenotypic variance. nComponents parameter used in this analysis was 3, with 10-fold cross-validation. While performing any downstream analysis around enrichment of identified analytes, only those that had correlation coefficient ≥ 0.5 were selected. This correlation threshold of the final model across selected analytes is a moderate conservative heuristic that was used to achieve a performance evaluation of AUC > 0.7 on average across each component with *p*-value < 0.05, to find a strong network of multi-omics analytes that could discriminate the categorical outcomes (S vs. As). This is also done while testing the data taking into consideration the DIABLO vignette and advises from the developers who have benchmarked this method and are continuously maintaining it at (https://mixomics.org/).

### Detailed multi-omics data integration and statistics

A systematic strategy with DIABLO was followed, applying the best practise from their vignette and their technical forum as suggested by the developers of the method, who have done extensive benchmarking of it previously [29] for the version of package used (version 6.25.1). Specifically, we first performed parameter optimization to identify what is the best nComponent metric with a number of samples and omics type we have as part of our study design. Usual rule of thumb is n-1 as advised by DIABLO developers, but we also tested it briefly, where we observed that using “centroid distance” the overall balanced error rate hit a plateau at 3 components within our design. Next, we systematically tested 0%,10%, 20%, 30% and 40% contribution of each omics layer in not only classifying our categorical variable (As vs S), but also helping assess pairwise correlation across omics layers for optimal feature selection using the nComponent as 3. Here we used M-Fold cross-validation of 10, while distance measurement was “centroid distance”. While doing this assessment, again is was observed that at 30% contribution, the balanced error rate (< 28% across all 3 nComponents) and the pairwise correlation coefficient between omics layers (e.g. metabolomics vs proteomics or proteomics vs. transcriptomics or transcriptomics vs. metabolomics) emerged as the best fit (minimum 30%) at an average. For any other contributions, there was a possibility of over or underfitting. Finally, this was used to run the *block.splsda* with a weighted design matrix of 30% contribution across omics types that can discriminate and explain the phenotypic variance with nComponents parameter, which in this analysis was set to 3, with 10-fold cross-validation and nrepeat parameter = 10 (the number of times the cross-validation will be repeated) for each Combination (1,2,3), reflective of peripheral circulation, local disease site and local plaque with peripheral circulation. This provided a list of transcripts, proteins and metabolites for each Combination, where we could further evaluate correlation coefficient across transcripts, proteins and metabolites alongside AUC values with a *p*-value < 0.05.

### Target mining and online bioinformatic tools

A list of all significantly differentially regulated molecules/analytes (genes, transcripts, metabolites or proteins) after corrections for multiple comparisons, was assembled from single- and multi-omics analyses. This list contained originally *n* = 268 analytes from single-omics and *n* = 111 from multi-omics. The list was then enriched with information from public databases on e.g. tissue-wide expression and localisation (GTex, HPA), plaque scRNAseq cellular fractions (PlaqView), druggability and epigenetic information (Enrichr), PubMed literature mining related to atherosclerosis/CVD, or data from animal models of atherosclerosis/CVD. The StringDB database was used to build protein-protein interaction networks for targets of interest.

Selected targets were checked for gene/protein-disease association with CVD traits using OpenTargets platform [31]. The platform provides evidence and global association score on user-defined targets to diseases of interest like atherosclerosis, coronary artery disease, heart failure or cardiovascular diseases in general. These scores can be further prioritized based on the strength of evidence, for hypothesis generation and drug development efforts.

### Correlation network analyses and visualization

Network analyses were performed by tools and databases (omicsnet 2.0, metaboanalyst 5.0, metabolon annotation list). Network diagrams were drawn in Cytoscape ver 3.8.2 and hubs identification by cytoHubba (Cytoscape Plugin). In more detail, we employed two complementary network strategies to reveal molecular interactions from different perspectives:

Evidence-based networks represent interactions that are known or highly predicted from accumulated biological knowledge in public databases. These databases are constructed prior to and independently of our specific dataset. The nodes (molecules) and edges (interactions) are derived from experimental evidence, curated pathways, or text-mining results. In this framework, the significance is not tested against a random network for its entire structure (as it is a repository of knowledge), but rather for the enrichment of our specific molecular list within this network. This is typically assessed using a hypergeometric test, which calculates whether the overlap between our gene list and a specific pathway/module is statistically significant compared to what would be expected by chance.

Correlation-based networks are entirely data-driven and constructed *de novo* from our experimental dataset. The edges are defined by statistical correlations between the expression/abundance levels of molecules across all samples. This approach can reveal co-regulated modules specific to our experimental condition, which may not yet be captured in existing knowledge bases. The key question of how these networks differ from chance is addressed by applying strict statistical thresholds to the correlations. We used a permutation-based or false discovery rate (FDR) approach to ensure that only edges with a correlation strength significantly greater than that expected in a random dataset were included.

This dual approach allowed us to prune the initial correlation-based results and extend them into the evidence-based methods, to produce more biologically relevant insights with functional connections and make relevant hypothesis. In more technical detail, networks were constructed in the following way:

#### Evidence-based networks

##### Input

The list of significant molecules from analysis by DIABLO.

#### Toolchain and parameters

##### MetaboAnalyst 5.0

Used for metabolite-metabolite interactions. The “Pathway Analysis” and “Network Explorer” modules were employed with the KEGG global metabolic network as the reference database. Default parameters were used, which map metabolites to their known biochemical pathways and reactions.

##### STRING (v11.5)

Used for protein-protein interactions (PPI). We used the default parameters with a medium confidence score threshold of > 0.4, which is a widely accepted benchmark balancing coverage and accuracy. Interaction sources were restricted to “Experiments,” “Databases,” and “Co-expression” to ensure evidence quality.

##### OmicsNet (v2.0)

Used to integrate gene-metabolite interactions and create a multi-omics context. We used the default “Comprehensive Network” setting, which queries several underlying databases.

Network Integration: The network files (in .graphml or .sif format) from the above tools were imported into Cytoscape. The networks were merged using Cytoscape’s built-in “Merge” function based on common node identifiers to create a unified evidence-based network.

#### Correlation-based network

##### Input

The quantitative data matrix (gene/protein expression values, metabolite intensities) across all samples.

##### Tool and statistical tests

The network was built within Cytoscape using the Expression Correlation plugin.

##### Correlation method

We selected the Spearman’s rank correlation coefficient due to its robustness against non-normally distributed data.

##### Statistical Thresholds (to address “difference from chance”)

To ensure only statistically significant edges were retained, we applied a dual-threshold approach:

- Correlation Coefficient Threshold: |ρ| > 0.5 (a moderately strong correlation threshold).
- Significance Threshold: Adjusted *P*-value < 0.05. The *P*-values for each correlation were calculated by the plugin and adjusted for multiple testing using the Benjamini-Hochberg False Discovery Rate (FDR) method.

The network was constructed using only edges that passed both filters.

#### Identification of hub nodes

For both the final evidence-based and correlation-based networks, hub nodes were identified using the cytoHubba plugin for Cytoscape. We utilized a combination of topology-based algorithms for robust identification, including Maximum Neighborhood Component (MNC), Degree, and Maximal Clique Centrality (MCC). Taking the consensus of top-ranked nodes from multiple methods increases confidence in the identified hubs. The top nodes from each algorithm were considered, and the most frequently occurring nodes across algorithms were defined as the key hubs.

### Joint enrichment analyses and visualization

Enrichment analysis was performed using clusterProfiler v4.8.2 based on databases (metabolon annotation list, KEGG, SMPDB, GO, Hallmark, Reactome pathways, WikiPathways). Joint enrichment analysis was performed using MetaboAnalyst 5.0; clusterProfiler v4.8.2, each leveraging Over-Representation Analysis (ORA) based on specific databases (KEGG, SMPDB). We meticulously calculated *p*-values and q-values, retaining only those results with (*P* < 0.05) and (Q < 0.1) to ensure statistical significance and control for multiple testing. R package ggplot2 v3.3.2 was used to create bubble plots, scatter plots, and boxplots. R/Bioconductor package ComplexHeatmap v2.2.0 was used to create all heatmaps. Venn diagrams were generated using the online tool InteractiVenn.

Beyond assessing statistical significance, we further enhanced joint enrichment analysis by incorporating a Z-score calculation to predict the up- or down-regulated status of the enriched pathways. The Z-score allowed to infer the activation or inhibition state of each enriched pathway, thereby providing a more comprehensive functional interpretation of joint omics data. The Z-score for each enriched pathway was calculated based on the number of up-regulated and down-regulated features (genes or metabolites) within that pathway. A commonly used formula for this enrichment score (ES) or Z-score is:

$$\:\boldsymbol{Z}\boldsymbol{s}\boldsymbol{c}\boldsymbol{o}\boldsymbol{r}\boldsymbol{e}=(\boldsymbol{u}\boldsymbol{p}-\boldsymbol{d}\boldsymbol{o}\boldsymbol{w}\boldsymbol{n})/\sqrt{\boldsymbol{c}\boldsymbol{o}\boldsymbol{u}\boldsymbol{n}\boldsymbol{t}}$$

Where ‘up’ refers to the number of significantly up-regulated features in the pathway, ‘down’ refers to the number of significantly down-regulated features in the pathway, and ‘count’ is the total number of significant features (up-regulated + down-regulated) in the pathway. A positive Z-score indicates that the pathway is predominantly up-regulated, while a negative Z-score suggests it is largely down-regulated.

With respect to more technical details of the joint enrichment analyses, the core statistical test underlying this analysis in both MetaboAnalyst 5.0 and clusterProfiler is the hypergeometric test. This test evaluates whether the number of specific genes or metabolites observed in a given pathway within our dataset is significantly higher than what would be expected by random chance.

#### MetaboAnalyst 5.0

For analyses primarily based on the KEGG database, MetaboAnalyst 5.0 employs the hypergeometric test to calculate enrichment *p*-values for identified metabolites or genes within specific pathways.

#### ClusterProfiler v4.8.2

The enricher function within clusterProfiler also utilizes the hypergeometric test. A key advantage of enricher is its flexibility in supporting custom databases. We leveraged this capability by downloading the SMPDB database from https://smpdb.ca/downloads and constructing a custom annotation file, which allowed us to perform ORA against SMPDB pathways.

#### *P*-value calculation

The *p*-value, derived from the hypergeometric test, quantifies the probability of observing at least the given number of hits in a pathway by random chance. A smaller *p*-value indicates stronger evidence against the null hypothesis of no enrichment.

#### Q-value calculation

To address the issue of multiple hypothesis testing inherent in pathway enrichment analysis, we calculated q-values. Q-values, also known as adjusted *p*-values or False Discovery Rate (FDR), control the expected proportion of false positives among the pathways deemed significant. Both MetaboAnalyst 5.0 and clusterProfiler typically use the Benjamini-Hochberg (BH) method for FDR adjustment.

#### Thresholds for significance

We strictly adhered to established thresholds, retaining only those enrichment results where the *p*-value was less than 0.05 (*P* < 0.05) and the corresponding q-value was less than 0.1 (Q < 0.1). This ensures that only robust and statistically significant enrichments contribute to our findings.

#### Data combination and plot generation

The “combination” for plot generation involved integrating these multi-faceted results from both MetaboAnalyst 5.0 (for KEGG-based enrichments) and clusterProfiler (for SMPDB-based enrichments). After filtering statistically significant pathways (*P* < 0.05), (Q < 0.1), the data was consolidated. The final figures were constructed by aggregating and visualizing these independently identified and directionally characterized pathways.

This approach allowed us to present a nuanced view of the perturbed biological processes, highlighting not only their statistical significance but also their predicted directional changes (up-regulated or down-regulated status) across the different omics layers and databases.

### Computed tomography angiography (CTA) and image analysis

Carotid CTAs was performed pre-operatively as previously described [32, 33]. Reconstructed images (0.625 mm) were analysed by trained observers using the ElucidVIVO^®^ (Elucid Bioimaging Inc., Boston, MA) software as previously described [32–34]. In brief, the external carotid artery was excluded from analyses and the lumen and wall of the common and internal carotid artery evaluated automatically where the software creates 3D segmentations with voxel-wise discrimination of intraplaque tissue types including lipid-rich necrotic core (LRNC), calcification (CALC), intraplaque haemorrhage (IPH), and extracellular matrix (MATX, representing plaque tissue not identified as any of the previous components). The proportion of these components relative to the total wall volume was quantified (VolProp) together with structural features such as wall volume plaque burden (proportion of total vessel volume or area occupied by the plaque), minimal fibrous cap thickness (shortest distance from the edge of the LRNC to the lumen in microm), and degree of stenosis (NASCET).

### Medication association analyses

The association of gene expression of top targets with medications in BiKE were calculated using a linear regression model given by:

$$\:\boldsymbol{G}\boldsymbol{e}\boldsymbol{n}\boldsymbol{e}\:\boldsymbol{e}\boldsymbol{x}\boldsymbol{p}\boldsymbol{r}\boldsymbol{e}\boldsymbol{s}\boldsymbol{s}\boldsymbol{i}\boldsymbol{o}\boldsymbol{n}=\:{\boldsymbol{\beta\:}}_{0}+\:{\boldsymbol{\beta\:}}_{1}\boldsymbol{*}\boldsymbol{m}\boldsymbol{e}\boldsymbol{d}\boldsymbol{i}\boldsymbol{c}\boldsymbol{a}\boldsymbol{t}\boldsymbol{i}\boldsymbol{o}\boldsymbol{n}+\:{\boldsymbol{\beta\:}}_{2}\boldsymbol{*}\boldsymbol{S}\boldsymbol{e}\boldsymbol{x}+\:{\boldsymbol{\beta\:}}_{3}\boldsymbol{*}\:\boldsymbol{A}\boldsymbol{g}\boldsymbol{e}+\:\boldsymbol{\epsilon}$$

where ***β***_***0***_, ***β***_***1***_, ***β***_***2***_ and ***β***_***3***_ are effect sizes for the corresponding predictor variables and ***ε*** is the error term. As described in the equation above, the association is adjusted for age and sex of the patients as confounders in the analysis.

### Survival analyses

The incidence for outcomes was plotted with Kaplan-Meier curves, with differences tested by log rank test. Survival curve was plotted using the number of myocardial infarctions (MI), ischemic strokes (IS), major cardiac and cerebral events (MACCE) and all-cause death at each unique time point for a period of 15 years after the patients have undergone carotid endarterectomy. To perform survival analysis, the patients were stratified according to the 25th quartile (low) and 75th quartile (high) of the relevant analyte levels. In more technical details, the associations of the gene expression with MACCE, MI, stroke and death were calculated using a Kaplan-Meier survival estimator model given by:

$$\:\widehat{\boldsymbol{S}}\left(\boldsymbol{t}\right)=\:\prod\:_{{\boldsymbol{t}}_{\boldsymbol{i}}\le\:\boldsymbol{t}}(1-\frac{{\boldsymbol{d}}_{\boldsymbol{i}}}{{\boldsymbol{n}}_{\boldsymbol{i}}})$$

where **t**_**i**_ are the ordered event times, **d**_**i**_ is the number of events (MACCE, MI, stroke or death) at time **t**_**i**_ and **n**_**i**_ is the number of individuals at risk just prior to time **t**_**i**_.

## Results

### Study design and workflow

**Fig. 2:**
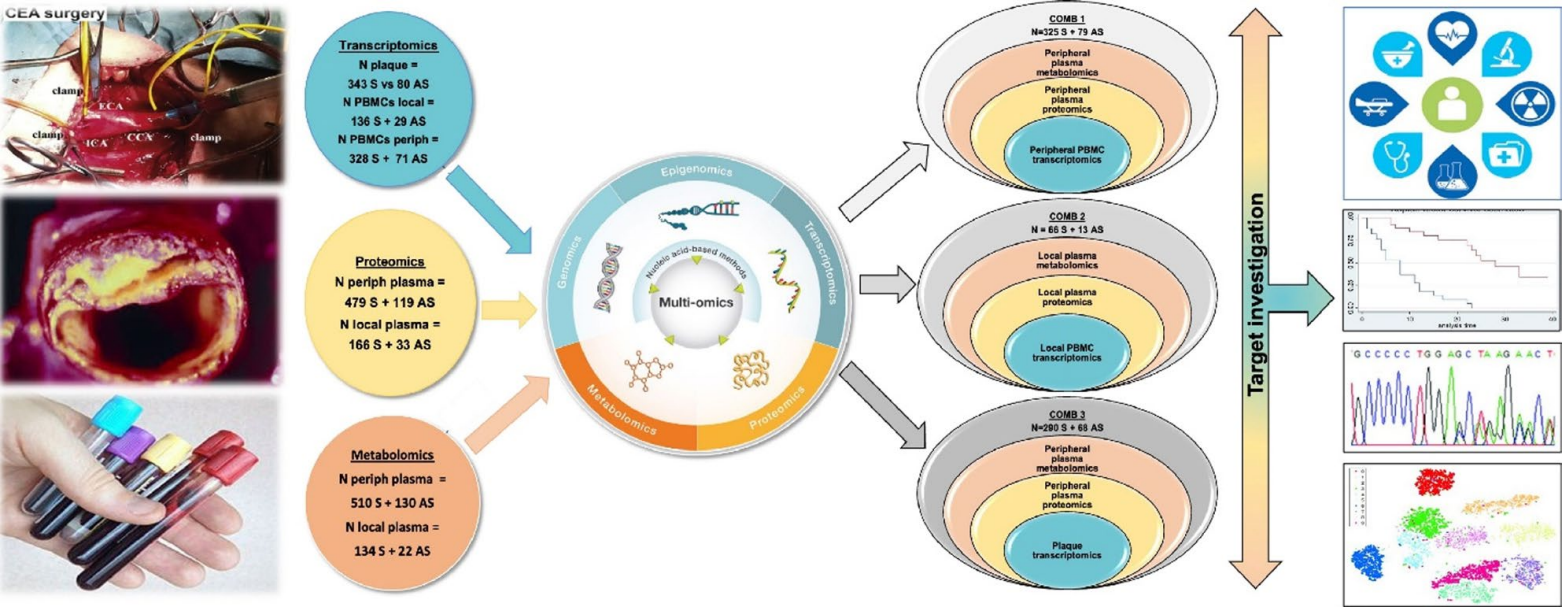
Illustration of the study design and multi-omics integration workflow. Samples for the Biobank of Karolinska Endarterectomy (BiKE) were successively collected at carotid endarterectomy (CEA) surgery since 2002, covering the whole region of Stockholm, Sweden. Plaques, PBMCs and plasma were profiled using large-scale, state-of-the-art omics technologies with transcriptomics by RNAseq, proteomics by OLINK and metabolomics by LC-MS/MS. Single-omics analyses were performed in each layer, comparing samples from symptomatic vs. asymptomatic patients. Multi-omics integration by DIABLO tool was performed on matched data from patients, coupling orthogonally peripheral (Combination 1) or local (Combination 2) sites, as well as an intersection of both (Combination 3). Top analytes from each Combination passing the correction for multiple comparisons, were investigated further on individual level by interrogation of clinical, epidemiological, follow-up, genetic data from the same patients as well as public scRNAseq datasets and database mining. S-symptomatic, AS-asymptomatic, PBMCs-peripheral blood monocytes

Biomaterial from atherosclerosis patients consisted of matched plaques, PBMCs and peripheral as well as “local” plasma samples, retrieved in the proximity of the lesion. The study was conducted in 3 major stages (Fig. 2), all stratified by comparisons between S vs. AS patients to identify molecular signatures of atherosclerotic instability and incorporating sex and age adjustments wherever applicable. The first stage consisted of large-scale single-omics data analyses (plaque and PBMCs transcriptomics, proteomics and metabolomics of plasma sampled form peripheral circulation or locally from the disease site). In the second stage, multi-omics was designed to integrate 3 combinations of datasets separately, where Combination 1 integrated data from peripheral circulation to identify potential biomarkers of atherosclerotic instability; Combination 2 integrated data from the local disease site, previously shown to be enriched with lesion-derived analytes [11]; and Combination 3 integrated data from plaques and peripheral circulation with an aim to detect molecular signatures in blood that originate from the culprit lesion. In the third stage, targeted translational analyses were performed for the top candidates from multi-omics integrations, associating their plaque and/or plasma levels with cell types, clinical biochemistry parameters, medication and outcomes in patients. Figure S1 shows the data generated for this study.

### Single-omics analyses comparing S vs. AS patients

#### Transcriptomics reveals enrichment of epithelial-to-mesenchymal transition, angiogenesis, inflammation and cholesterol metabolism in plaques and PBMCs from S vs. AS patients

After quality control, analysis was performed for *n* > 16 000 transcripts in a total of *n* = 423 plaques (343 S + 80 AS patients), *n* = 165 local PBMCs (136 S + 29 AS patients) and *n* = 399 peripheral PBMCs (328 S + 71 AS patients) (Figure S2). Differential transcript level analyses were performed comparing S vs. AS patients, with sex and age as covariates and correction for multiple comparisons, in PBMCs and plaque samples separately. This analysis resulted in 152 differentially regulated transcripts in plaques, 9 in local and 586 in peripheral PBMCs.

Among the top 20 significantly upregulated genes in plaques from S patients were e.g. CHI3L2, CXCL6, CLEC4G, MMP16, CFB and PI15 (Table S2). Gene set enrichment analysis showed that epithelial-to-mesenchymal transition, TNFa and IL6 signalling, IFNg response, hypoxia and angiogenesis, ECM reorganisation and bone remodelling, cholesterol homeostasis and glycolysis were some of the most important pathways in plaques. Functional associations among the significantly dysregulated genes were related to cell migration, tissue remodelling, actin binding. Most active associations were found at the basement membrane and actomyosin cytoskeleton, while interactions enriched in the network were related to focal adhesions and ECM (Figure S3).

Similar analyses in local PBMCs showed an upregulation of i.e. CCL2, GPNMB, MMP19, TREM2, IL10 and an enrichment of epithelial-to-mesenchymal transition, TNFa and IL6 signalling, IFNg response, coagulation, heme metabolism and angiogenesis, cholesterol homeostasis and glycolysis as important pathways (Table S3, Figure S3).

In peripheral PBMCs 102 transcripts were upregulated, such as LGMN, IGFBP4, SYNPO, PODXL, COL6A1, while again epithelial-to-mesenchymal transition, TGFB/WNT and IL2 signalling, coagulation, angiogenesis, cholesterol homeostasis and cell apoptosis processes were enriched. Functional interactions among the differentially regulated genes in both local and peripheral PBMCs included cartilage development and proteoglycans (Table S4, Figure S3).

#### Proteomics highlights enrichment of chemokine signalling in both local and peripheral plasma, while IL17 pathway was specifically upregulated locally

After quality control, analysis was performed for *n* = 442 proteins in a total of *n* = 598 peripheral (479 S + 119 AS patients) and *n* = 199 local plasma samples (166 S + 33 AS patients) (Figure S2). Differential protein levels analysis was performed comparing S vs. AS patients, with sex and age as covariates, in local and peripheral samples separately. After adjustments, 68 proteins were found to be dysregulated in peripheral and 36 in local plasma (Tables S5 and S6), of which *n* = 15 proteins were shared between the local and peripheral plasma (Figure S4A). Commonly dysregulated proteins included known atherosclerosis markers (i.e. CSF1, CDH5, FASLG, NOTCH3, IL6, IL6RN, EGFR, CXCL8, PLAUR), but also some less explored candidates (i.e. ICOSLG, PCOLCE, FUT5, CCL20, CA6, KITLG).

Enrichment analysis confirmed the overall upregulation of chemokine signalling and cytokine-receptor interactions in both local and peripheral plasma, while IL17 pathway was specifically enriched locally (Figure S4). Functional network coupling reinforced the notion that several of these proteins were also key drivers of the differential changes observed in both local and peripheral blood (IL6, EGFR, CXCL8), while others had a more prominent function in local (CSF1) or peripheral (TIMP1, CXCL12) disease sites, respectively (Figure S4).

#### Metabolomics shows upregulation of sphingomyelins and downregulation of bilirubin degradation products in association with symptomatology

After quality control, analysis was performed for *n* = 1011 metabolites in a total of *n* = 640 peripheral (510 S + 130 AS patients) and *n* = 156 local plasma samples (134 S + 22 AS patients) (Figure S2). Differential metabolite analysis was performed comparing S vs. AS patients, with sex and age as covariates, in local and peripheral plasma samples separately. In peripheral plasma, there were *n* = 213 metabolites that were found to be nominally dysregulated and in the local plasma *n* = 76 (Tables S7 and S8). Totally *n* = 31 metabolites were commonly dysregulated in both local and peripheral plasma (). Notably, tryptophan betaine, Bilirubin and Bilirubin degradation products were downregulated, while various Sphingomyelins, glyco-beta-muricholate and mannonate were upregulated in S vs. AS local and peripheral plasma comparisons.

Enrichment analysis confirmed these results, reinforcing the increase in Sphingomyelins in both local and peripheral blood, while Bilirubin degradation products remained significantly repressed in peripheral blood (Figure S5).

### Multi-omics integration analyses

Multi-omics integration of BiKE datasets was next performed with a rationale to identify complex orthogonal relationships across molecular species/analytes associated with unstable carotid atherosclerosis. To this end, matched patient samples and analytes for each of the 3 multi-omics combinations (peripheral circulation, local disease site, intersection between plaque and peripheral site) were first identified (Figures S6 and S7).

#### Multi-omics data integration in peripheral circulation reveals FABP4, Sphingomyelins, IL6 and bilirubin degradation as key signatures associated with symptoms

**Fig. 3:**
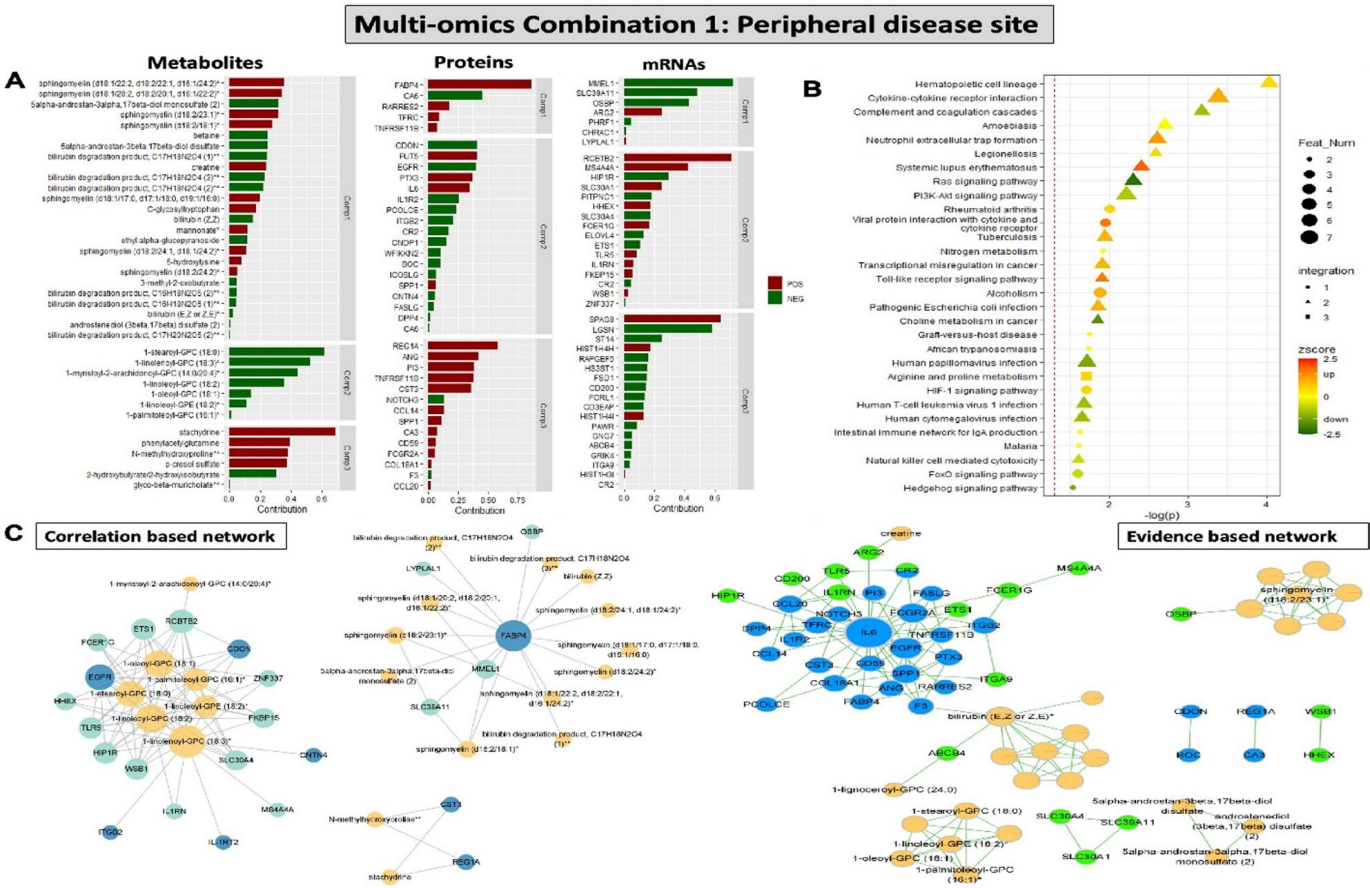
Multi-omics data integration around the peripheral disease site (Combination 1). **A** Contribution of various metabolites, proteins and mRNAs from relevant single-omics layers to the integrated multi-omics model with 3 components. Bars represent the contribution of each feature to the symptomatic patient phenotype. Direction of contribution indicated with color (red-positive, green-negative). **B** Joint enrichment analysis of all 3 omics layers in the multi-omics model. Circle indicates that the enriched term contains only one type of omics data, triangle represents terms containing 2 omics types, and square for 3 types. Direction of effect indicated with colour (green-to-red, legend). **C** Correlation based network (rho > 0.5) (to the left) and evidence-based network (to the right) of different analytical features from the multi-omics analyses. Color represents the type of omics data (blue, green and yellow referring to proteins, transcripts and metabolites respectively), size of the node represents the degree of significance. Feat Num-feature number

Contribution of various metabolites, proteins and transcripts from relevant single-omics layers (peripheral PBMC transcriptomics + peripheral plasma proteomics + peripheral plasma metabolomics, referred to as Combination 1) to the integrated multi-omics model in peripheral circulation was analyzed using the DIABLO tool and stratified according to symptomatology (Fig. 3A). The analysis showed that a signature composed of i.e. upregulation of various Sphingomyelins and downregulation of Bilirubin degradation metabolites, along with upregulation of FABP4, IL6, ANG, TNFRSF11 and SPP1 proteins, as well as upregulation of ARG2, SPAG8 with downregulation of MMEL1, OSBP, LGSN transcripts, was collectively enriched in the peripheral circulation of S patients. Joint enrichment analysis of all 3 omics layers in the multi-omics model, showed an enrichment of hematopoietic cell lineage pathways, arginine metabolism and inflammation, while Ras and PI3K/Akt signalling were repressed (Fig. 3B). Correlation based network analysis highlighted an association of FABP4 with various Sphingomyelins and Bilirubin degradation, while evidence-based network centered around IL6 interactome and Bilirubin degradation products (Fig. 3C).

#### Multi-omics data integration at the local disease site reveals Sphingomyelins, ANGPTL3, F11, ICOSLG, ITGB1, SOX11, MPZL1, TFPI as key signatures associated with symptoms

**Fig. 4:**
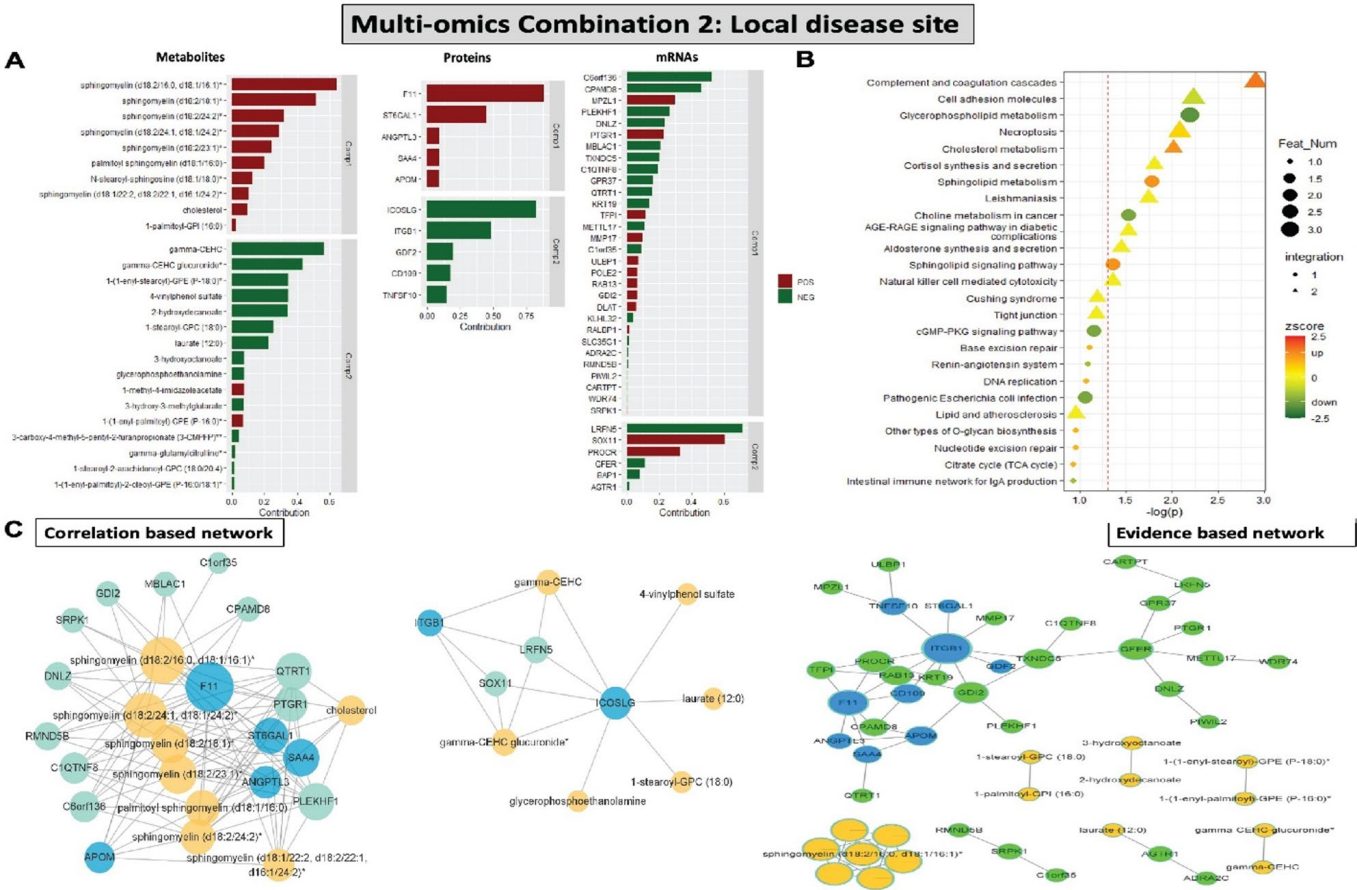
Multi-omics data integration around the local disease site (Combination 2). **A** Contribution of various metabolites, proteins and mRNAs from relevant single-omics layers to the integrated multi-omics model with 2 components. Bars represent the contribution of each feature to the symptomatic patient phenotype. Direction of contribution indicated with color (red-positive, green-negative). **B** Joint enrichment analysis of all 3 omics layers in the multi-omics model. Circle indicates that the enriched term contains only one type of omics data, triangle represents terms containing 2 omics types, and square for 3 types. Direction of effect indicated with colour (green-to-red, legend). **C** Correlation based network (rho > 0.5) (to the left) and evidence-based network (to the right) of different analytical features from the multi-omics analyses. Color represents the type of omics data (blue, green and yellow referring to proteins, transcripts and metabolites respectively), size of the node represents the degree of significance. Feat Num-feature number

Next, the contribution of various analytes from 3 omics layers to the integrated multi-omics model around the local disease site (local PBMC transcriptomics + local plasma proteomics + local plasma metabolomics, Combination 2) was also analyzed in connection to the presence of symptoms (Fig. 4A). Again, the analysis highlighted strong upregulation of various Sphingomyelins and showed that a signature composed from i.e. Sphingomyelin metabolites, along with the dysregulation of F11, ST6GAL1, ANGPTL3, ICOSLG, ITGB1 proteins, as well as dysregulation of SOX11, MPZL1, TFPI, PTGR1 transcripts, was collectively enriched at the local disease site in S patients. Joint enrichment analysis of omics layers in this model, demonstrated coagulation, cholesterol metabolism and necroptosis as the most prominent pathways (Fig. 4B). Correlation based network analysis highlighted a direct association of F11 and ANGPTL3 with various Sphingomyelins and cholesterol, while evidence-based network centered around the ITGB1, F11 and ANGPTL3 interactomes (Fig. 4C).

#### Multi-omics data integration combining plaque and peripheral disease site reveals Sphingomyelins, fatty acids, bilirubin degradation products, complement components, FABP4 and IL6 as key signatures associated with symptoms

**Fig. 5:**
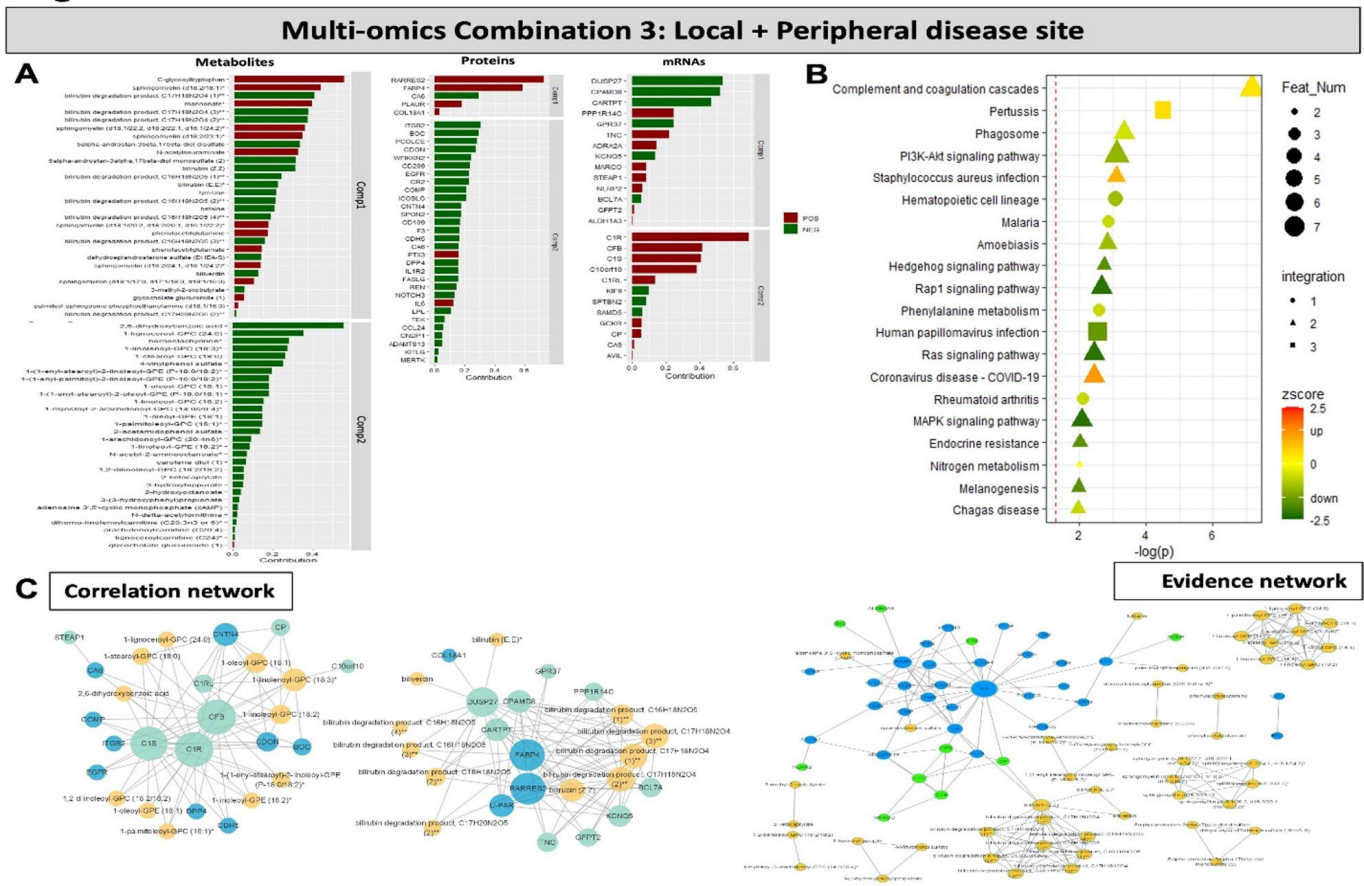
Multi-omics data integration combining local and peripheral disease site (Combination 3). **A** Contribution of various metabolites, proteins and mRNAs from relevant single-omics layers to the integrated multi-omics model with 2 components. Bars represent the contribution of each feature to the symptomatic patient phenotype. Direction of contribution indicated with color (red-positive, green-negative). **B** Joint enrichment analysis of all 3 omics layers in the multi-omics model. Circle indicates that the enriched term contains only one type of omics data, triangle represents terms containing 2 omics types, and square for 3 types. Direction of effect indicated with colour (green-to-red, legend). **C** Correlation based network (rho > 0.5) (to the left) and evidence-based network (to the right) of different analytical features from the multi-omics analyses. Color represents the type of omics data (blue, green and yellow referring to proteins, transcripts and metabolites respectively), size of the node represents the degree of significance. Feat Num-feature number

Finally, the contribution of various analytes from relevant single-omics layers to the integrated multi-omics model at the intersection between peripheral and local disease site (plaque transcriptomics + peripheral plasma proteomics + peripheral plasma metabolomics, Combination 3) was analyzed and stratified by symptomatology (Fig. 5A). Once again, the analysis showed that a signature composed from i.e. upregulation of various Sphingomyelins and downregulation of Bilirubin degradation metabolites and many fatty acids, along with the upregulation of RARRES2, FABP4, PLAUR, IL6 and downregulation of PCOLCE, COMP, ICOSLG proteins, as well as upregulation of TNC, MARCO, C1R, C1RL, C1S, CFG transcripts, was enriched in S patients. Joint enrichment analysis of all 3 omics layers in the multi-omics model, highlighted various infectious pathways (Fig. 5B). A correlation-based analysis highlighted two main networks: one *via* a direct FABP4 association with RARRES2 and various Bilirubin degradation products, and another *via* several complement components (C1S, C1R, CFB) and various fatty acids. An evidence-based network gave more dispersed results, but it could be noted that the IL6 interactome linked with Bilirubin degradation products *via* complement components, while Sphingomyelins and fatty acids formed separate interactomes (Fig. 5C).

#### Functional comparison of all three integrated multi-omics combinations points to ICOSLG and sphingomyelins as common factors in local and peripheral disease sites

**Fig. 6:**
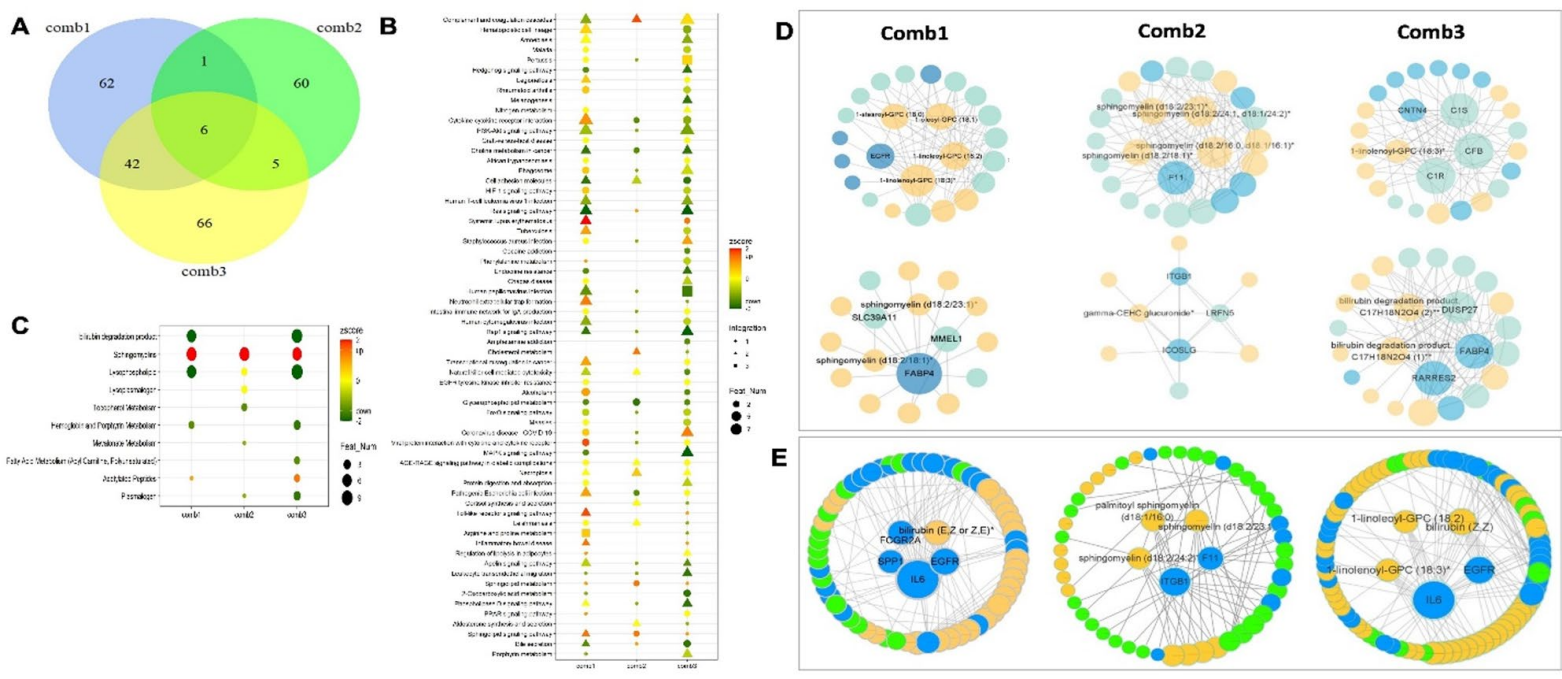
Functional comparison of all three integrated multi-omics combinations. **A** Venn diagram showing numbers of analytes identified in each multi-omics combination, including a list of 6 overlapping analytes (1-stearoyl-GPC (18:0), ICOSLG, sphingomyelin d18:1/22:2, d18:2/22:1, d16:1/24:2), sphingomyelin (d18:2/18:1), sphingomyelin (d18:2/23:1) and sphingomyelin (d18:2/24:1, d18:1/24:2)). **B** Joint and **C** metabolomic data enrichment analyses for all 3 combinations, illustrating the number of different integrations (triangle, square, circle), direction of effect (color green-to-red) and the number of features per pathway (size of the node). Correlation **D** or evidence-based **E** functional network analyses of the 3 multi-omics integration combinations. Metabolites in yellow, proteins in blue, transcripts in green. Feat Num-feature number, Comb-combination

An overlap of the 3 integration combinations stratified by symptomatology, contained 6 commonly discovered analytes across all multi-omics combinatorial analyses. These were ICOSLG, several Sphingomyelins and 1-stearoyl-GPC (Fig. 6A). A detailed list of analytes from various multi-omics integrations is shown in Table S9.

Joint gene set enrichment analysis revealed the importance of coagulation and necroptosis across all 3 combinations, but also activation of various immune processes and cholesterol metabolism as the most important pathways (Fig. 6B). Joint metabolomic analysis specifically confirmed the enrichment of Sphingomyelins and the repression of Bilirubin degradation products across all 3 integrations (Fig. 6C). Correlation- and evidence-based functional network analyses again highlighted the overall interplay among Sphingomyelins, coagulation factors and inflammation (Fig. 6D, E). In particular, network in Combination 1 (only peripheral plasma) was centered around IL6, EGFR, Bilirubin, SPP1, FABP4 and Sphingomyelins; network in Combination 2 (only local disease site) was centered around Sphingomyelins, ITGB1 and F11; while network in Combination 3 (the integration of plaque with peripheral plasma) centered again around IL6, EGFR and Bilirubin.

### Clinical, genetic and long-term follow-up studies for selected targets of interest

#### Analysis of selected targets with respect to cellular and morphological plaque features

We next assembled and interrogated the longer list of individual targets compiled from all single-omics analyses and multi-omics integrations with following criteria: (1) targets that showed individual significance in BiKE datasets, (2) those that repeatedly appeared in various analyses across the different Combinations and (3) those that were highlighted as key network drivers in different Combinations. The Table S10 shows the full list of approximately 380 targets, systematically enriched with functional and expression information from BiKE and various public databases.

From this list, BLVRA and BLVRB were selected as the key enzymes representing Bilirubin metabolism, which was highlighted in analyses of peripheral plasma along with FABP4 (Combination 1). ANGPTL3 was the gene that emerged from the local disease site studies (Combination 2), while ICOSLG and SMPD1 (a key enzyme in the Sphingomyelin metabolism) were selected as main genes from the intersection between local and peripheral disease sites (Combination 3). An *in-depth* BiKE analysis was performed for each of these top targets with respect to their expression levels across all datasets, association with plaque cells and morphological features, genetics, patient clinical chemistry parameters, medications, as well as future outcomes, to assess their translational potential.

**Fig. 7:**
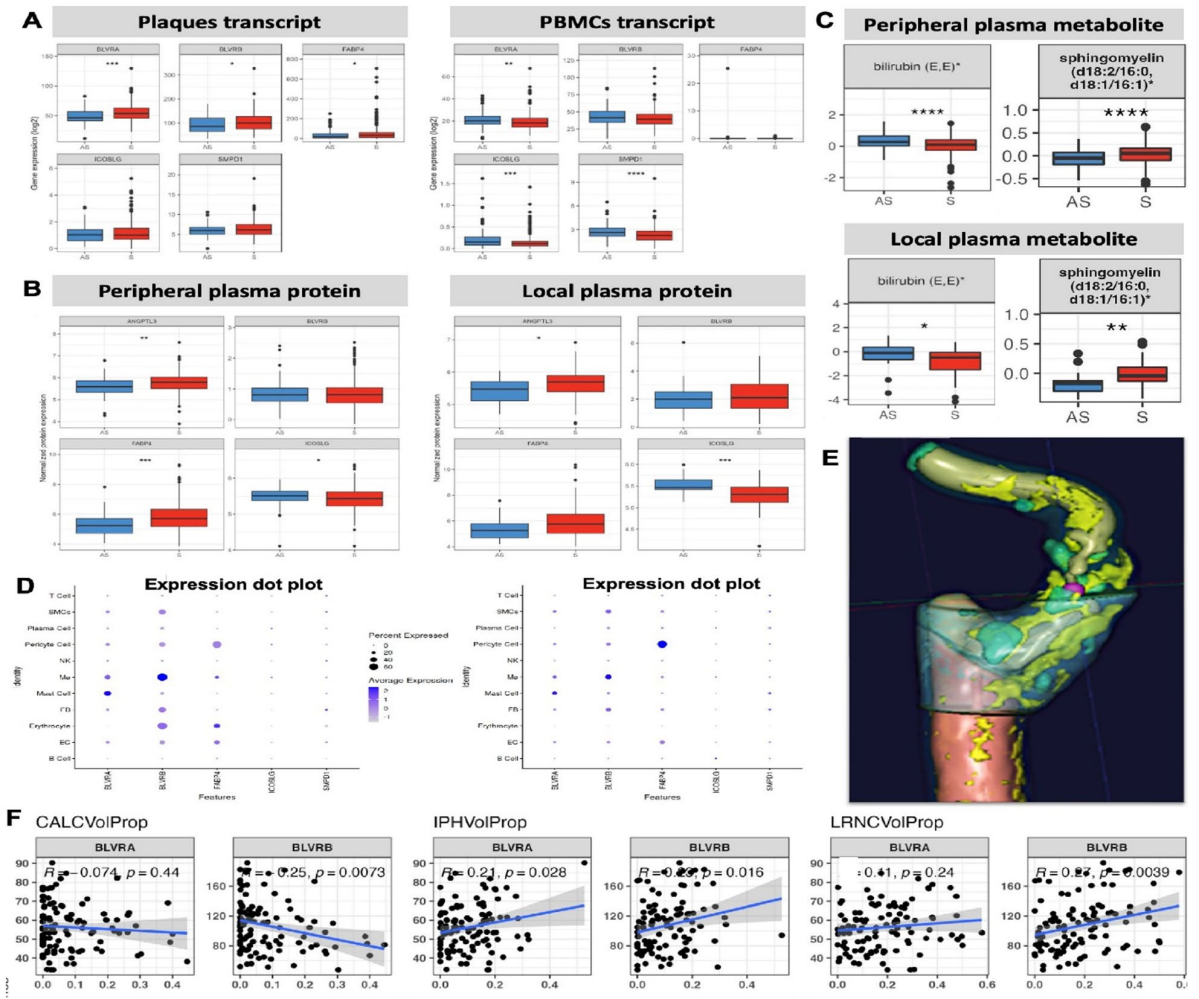
Expression analysis of selected targets of interest and association with plaque cellular composition and morphological features. **A** mRNA expression of selected targets of interest BLVRA, BLVRB, FABP4, ICOSLG and SMPD1 in plaque and PBMC transcriptomic RNAseq data comparing S vs. AS patients. ANGPTL3 expression was not detected. **B** Proteomic analysis of peripheral and local plasma comparing normalized levels of targets of interest in S vs. AS patients. **C** Analysis of metabolomic data from peripheral and local plasma comparing normalized levels of representative bilirubin and sphingomyelin metabolism targets in S vs. AS patients. **D** Carotid plaque scRNAseq data from *Alsaigh et al. 2020* (left) and *Wirka et al. 2019* (right) queried *via* the PlaqView open resource for cellular expression of selected targets. **E** An illustration of the computed tomography (CT) carotid plaque image analyzed with the ElucidVivo software. Colors indicate different features, i.e. lipids are yellow, calcification turquoise, matrix is blue, etc. **F** Correlation analyses were performed between the plaque RNA expression of selected targets and various quantified morphological features from diagnostic CTs (*n* = 160 patients). IPHVolProp-intraplaque hemorrhage volume proportion, CalcVolProp-calcification volume proportion, LRNCVolProp-lipid rich necrotic core volume proportion, S-symptomatic, AS-asymptomatic, PBMCs-peripheral blood monocytes

Significant upregulation of BLVRA and BLVRB transcripts was found in comparison of plaques from S vs. AS patients, and BLVRA was also dysregulated in PBMCs (Fig. 7A). Although plasma protein levels of these enzymes were not increased in this study (Fig. 7B), various components of the Bilirubin metabolism were detected in metabolomic analysis and all consistently showed downregulation in S vs. AS patients, both in local and in peripheral plasma (representative metabolites in Fig. 7C). FABP4 transcript was significantly upregulated in plaques from S vs. AS patients, consistent with FABP4 protein being significantly increased in both local and peripheral plasma from S individuals. A significant downregulation of ICOSLG transcript and protein levels was detected in PBMCs, local and peripheral plasma from S vs. AS patients. While ANGPTL3 transcript was below the detection levels in plaques or in PBMCs, protein levels were significantly increased in peripheral and local plasma from S vs. AS individuals. Finally, SMPD1 transcript was detected at low levels and downregulated in PBMCs from S vs. AS patients. Protein levels of this enzyme were not analyzed in plasma in this study, but various components of the Sphingomyelin metabolism were all consistently upregulated in both local and peripheral plasma from S patients (representative metabolites in Fig. 7C).

Additionally, utilizing public scRNAseq datasets from human carotid plaques [35, 36], we could further establish that BLVRA and BLVRB were both expressed in macrophages, as previously reported by us and others [11], but the expression of BLVRB was considerably higher and more broadly associated also with erythrocytes, fibroblasts, pericytes and SMCs in plaques, while BLVRA was found also in mast cells (Fig. 7D). FABP4 seemed to be mostly expressed by pericytes, which has not been investigated so far, and lower levels were detected in macrophages, erythrocytes and endothelial cells. ICOSLG and SMPD1 were again expressed at low levels and could not be definitively localized in plaque cells, while ANGPTL3 was confirmed as undetectable.

The association of selected targets with plaque morphology features was next assessed by correlating BiKE RNAseq data with quantification of plaque tissue components from image analyses of pre-operative CTAs, as previously described (Fig. 7E and F) [32–34]. Here, BLVRA and BLVRB significantly positively associated with total intraplaque hemorrhage volume proportion. BLVRB showed a positive association also with lipid rich necrotic core and a negative association with plaque calcification volume proportion. The other genes of interest did not show associations with plaque morphology in this analysis.

#### Association of selected targets with patient clinical biochemistry parameters

A detailed correlation analysis was performed between various blood parameters and transcript, protein or metabolite levels for selected targets (Figure S8). Here, the most consistent correlations in plaques and plasma were found for BLVRB, FABP4 and ICOSLG. Our analysis suggested a significantly positive association for measures of kidney function with FABP4 and ICOSLG. Plasma levels of FABP4 were also positively associated with inflammatory parameters such as CRP, leucocyte count and fibrinogen. Another positive association was found between Hb1ac levels, and levels of BLVRB, FABP4, ICOSLG. Moreover, there was a significantly positive association between plasma levels of ANGPTL3 and total serum cholesterol in patients.

With respect to metabolites, positive association was found between Bilirubin levels and Bilirubin degradation products, as expected (Figure S8). These metabolites were also consistently positively correlated to Hb levels and erythrocytes, but negatively to CRP, HbA1c and fibrinogen, and without correlations to lipid parameters. On the other hand, plasma levels of Sphingomyelins correlated positively with most lipid parameters (total cholesterol, LDL, HDL, except TG) and adiponectin levels, but negatively with Hb and HbA1C. These correlations suggest possible mechanistic dependencies among the targets of interest and classical, clinically used blood markers of various cells and processes.

#### Association of selected targets with medications

Information about relevant medications, i.e. lipid-lowering, anti-diabetic, anti-hypertensive and anti-thrombotic therapies, was available in BiKE for the majority of patients and was tested in association with the levels of various analytes in plaques, PBMCs and plasma (selected data shown in Figure S9). After adjustment for age and sex, significant results could be shown for the transcript expression of SMPD1, ICOSLG and BLVRB in peripheral PBMCs and treatment with anti-thrombotics, anti-hypertensives and in particular lipid-decreasers, where the association with ICOLSG was strongly significant. On protein level, anti-hypertensive medication showed an effect on FABP4 in both local and peripheral plasma. On metabolite level, anti-hypertensive medication also showed marginal association with plasma Bilirubin, while anti-diabetics had an impact on plasma Sphingomyelins. Of interest, plasma ANGPTL3 protein levels were not affected by any of the tested medications.

#### Association of selected targets with long-term survival of patients

Survival has been monitored for > 15 years in BiKE patients [30], with respect to the cumulative MACCEs (MI and ischemic stroke events), as well as all cause death. This allowed us to interrogate the association between levels of selected targets and adverse outcomes, which could suggest biomarkers of long-term risk in patients already treated with CEA.

**Fig. 8:**
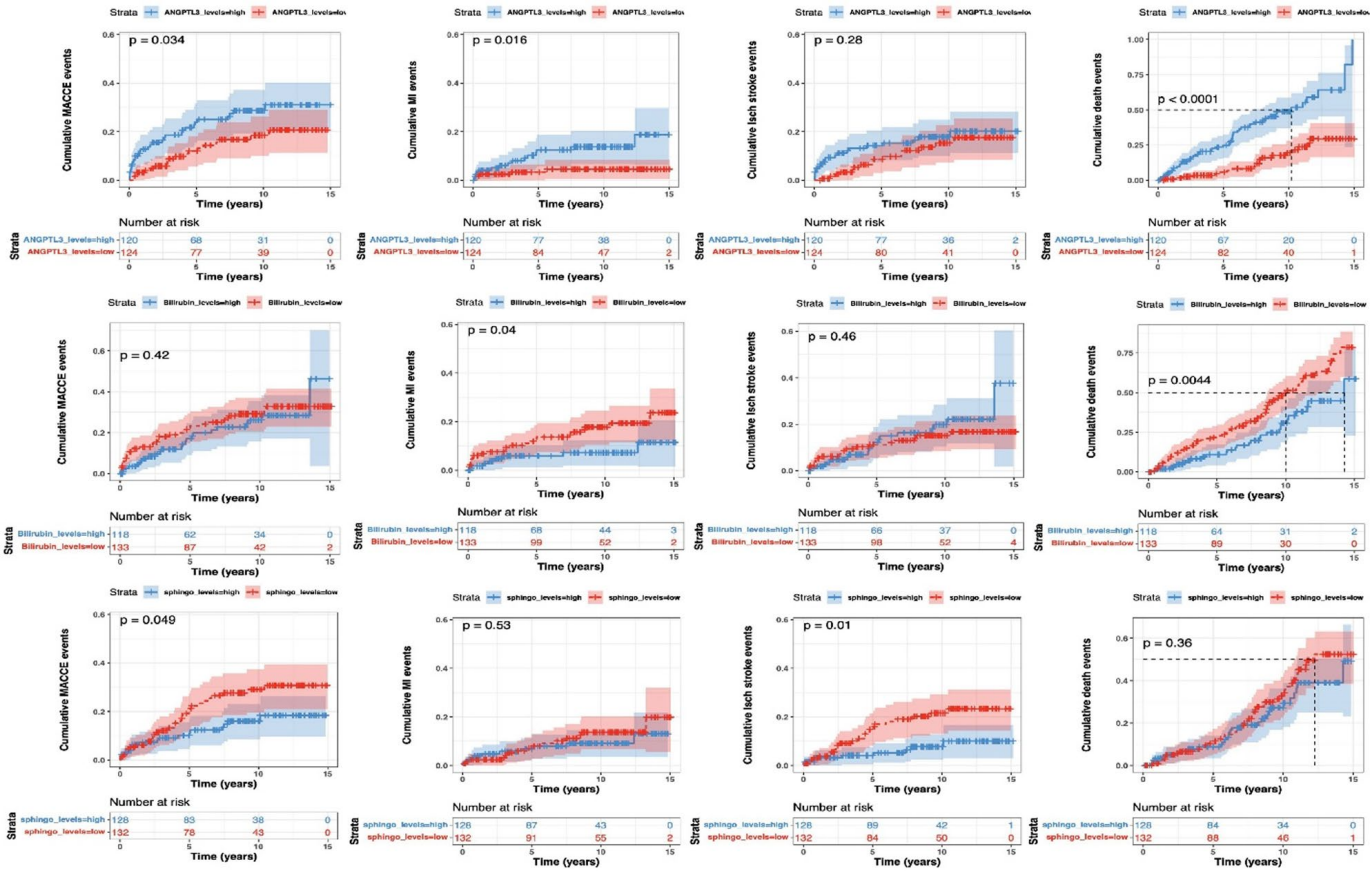
Cumulative Kaplan-Meier outcome estimates after carotid surgery. Plots illustrating survival of BiKE patients during the 15 years follow-up period after surgery, based on top vs. bottom quartile of relevant analyte levels in peripheral plasma. Both patients that were symptomatic and asymptomatic at surgery were included in the follow-up analysis. Each mark along the lines indicates an event, numbers at risk indicated in tables under the plots. Top raw shows plots for peripheral plasma ANGPTL3 protein levels. Middle raw shows plots for Bilirubin metabolite peripheral plasma levels, and bottom raw Sphingomyelin metabolite peripheral plasma levels. MACCEs-major adverse cardio- and cerebro-vascular events; MI-myocardial infarction

Analysis showed that patients with higher peripheral plasma protein levels of ANGPTL3 (top quartile) suffered from significantly more MACCEs and all cause death, showing an overall vulnerable profile (Fig. 8). Lower (bottom quartile) Bilirubin metabolite peripheral plasma levels were indicative of MIs and all cause death (Fig. 8), but lower plaque BLVRB expression was associated with less MACCEs and MIs (Figure S10). Of note, BLVRA did not show any significance in this analysis. Lower levels of various Sphingomyelins in peripheral plasma systematically associated with more MACCEs and ischemic strokes (Fig. 8). Higher expression of ICOSLG in plaques was linked with poorer survival with respect to MACCEs and ischemic stroke, but FABP4 expression did not show any significance in these analyses (Figure S10).

#### Prioritized targets show genetic linkage with cardiovascular disease traits

Finally, previously prioritized candidates (FABP4, BLVRA, BLVRB, SMPD1, ICOSLG, ANGPTL3) were extended with other top analytes from each of the multi-omics integrations (F11, IL6, ITGB1, MPZL1, SOX11, TFP, C1R, C1S, CFB, EGFR, SPP1), together forming a list of 17 candidates selected for genetic association analyses. We tested for target-CVD associations using the public OpenTargets platform, a powerful tool for drug discovery that, by integrating diverse data sources like genomics, proteomics, literature, chemical databases and clinical trials, can identify genes potentially involved in cardiovascular conditions. It was noteworthy that 5 out of these 17 targets showed the overall global association score > 0.4 for CVD (on a scale of 0–1, with 1 being the strongest association), with F11, C1S, EGFR, IL6, ANGPTL3 being the top on this list. Additionally, 4 out of these top targets (F11, C1S, EGFR and IL6) showed strong contribution of genetics in CVD (Table S11). Of note, we tested for traits like atherosclerosis, carotid atherosclerosis and coronary artery disease in a similar way, but such specific analysis did not reveal strong target-disease associations.

## Discussion

Single- and multi-omics analyses were performed on data from a large biobank of carotid atherosclerosis, with focus on instability as defined by patient symptomatology. Key pathways and molecular signatures of unstable disease were confirmed, such as coagulation, necroptosis, immune activation, cholesterol metabolism, while less explored ones were also highlighted, i.e. ICOSLG, heme- and Bilirubin degradation and Sphingomyelin metabolism.

The single-omics analyses performed in this study using state-of-the-art technologies, are some of the most comprehensive ever reported in the field, illuminating key molecular features of unstable atherosclerosis. Plaque transcriptomics confirmed important pathways in plaque instability such as epithelial-to-mesenchymal transition, TNFa and IL6 signalling, IFNg response, hypoxia and angiogenesis, ECM reorganisation and bone remodelling, cholesterol homeostasis and glycolysis. Many of these pathways were also found in PBMC transcriptomics, in addition to coagulation, heme-metabolism and angiogenesis that seemed more specifically associated with circulating cells. Plasma proteomics confirmed the overall upregulation of chemokine signalling and cytokine-receptor interactions, while IL17 pathway was particularly enriched in local plasma retrieved from the vicinity of plaque at surgery. Plasma metabolomics revealed an increase in Sphingomyelins in both local and peripheral samples, while Bilirubin metabolites were significantly repressed in peripheral plasma. The metabolomics dataset is novel and potentially of high relevance for future studies in the field, considering that atherosclerosis is linked with systemic metabolic dysregulation [37]. The rapid growth in metabolomics platforms has only just begun to provide insights into the new pathophysiological pathways for CAD, cardiomyopathies, stroke, and diabetes [38–42]. Moreover, the power of pharmacometabolomic research has recently been demonstrated, when it was reported that baseline plasma levels of a single metabolite 2-hydroxyvaleric acid, can discriminate between responders and non-responders to statins [42]. Nevertheless, single-omics technologies remain insufficient in capturing the complex intricacies of human disease biology.

In recent years, multi-omics has shown promise for providing more robust subsystem classifiers and help in elucidating interactome interplay around the phenotype of interest [28]. The complex, multi-factorial nature of CVD makes it difficult to recapitulate the key molecular drivers and signalling pathways without system level approaches. This notion is strongly emphasised in our study, as both well-known and novel molecular targets and mechanisms appeared from each of the three different multi-omics integration combinations. Integration of data in Combination 1 from peripheral circulation solely was intended to yield potential biomarker discoveries. Indeed, IL6 as one of the top targets that emerged from this integration, is widely supported by numerous previous reports associating it with adverse cardiovascular outcomes, both in subjects with and without a history of CVD [43]. Combination 2 integrated only data from the local disease site that we have previously shown to be enriched with lesion-derived analytes [11]. Here, candidates such as F11 and ANGPTL3 appeared, possibly reflecting their association with coagulation/hemorrhage, and lipid accumulation in plasma in the vicinity of the lesion, respectively [44, 45]. Finally, Combination 3, integrating plaque with peripheral circulation data aimed to enrich our results for blood signatures specifically reflecting carotid plaque phenotype. This analysis showed that there was at least a portion of molecular signatures related to the lipid metabolism (i.e. FABP4, Sphingomyelins), coagulation (C1R), inflammation (IL6), but also signatures of hemoglobin degradation (Bilirubin), which were detectable in peripheral plasma and possibly reflect instability processes associated with intraplaque hemorrhage (IPH). Together, these well-known and novel markers constitute prioritized candidates for future development of biomarkers and pharmacotherapy.

Here, we validated the identified omics targets by performing translational analyses using patient clinical data. Our results showed that various cell types could be the sources of FABP4 in both plaques and plasma. FABP4 increase in plasma could potentially reflect systemic inflammation, diabetes and kidney dysfunction, as well as hypertension, indicated by the finding that anti-hypertensive medication was associated with plasma FABP4 protein levels. From these observations, it is possible that measurement of FABP4 levels could be used to improve patient phenotyping. Previous studies have already reported increased expression of FABP4 in carotid plaques, association to adverse events on short-term follow up, and suggested that it could be a key factor connecting vascular lipid accumulation with inflammation [46, 47].

ICOSLG is a ligand for the T-cell-specific surface receptor ICOS, whose expression is enhanced by TNF. It acts as a costimulatory signal for T-cell proliferation and cytokine secretion and serves an important role in mediating local tissue responses to inflammatory conditions [48]. In our study, ICOSLG was generally repressed in plaques, PBMCs and plasma from S patients on cohort level, but in those patients with a relatively higher expression in their plaques, it was associated with future adverse events. In PBMCs, its expression was strongly affected by lipid-lowering medication. It has previously been shown that ICOSLG antibodies reduced the development of atherosclerosis coupled to the formation of tertiary lymphoid organs [48]. ICOS/ICOSLG pathway has been suggested as a promising target for a new wave of immunotherapies, confirmed in our studies.

ANGPTL3 is a target that was solely identified in plasma, enriched in association with S disease and blood levels of all major lipid fractions, where high levels of ANGPTL3 were a strong predictor for poor long-term survival. ANGPTL3 is a factor secreted by the liver that inhibits LPL and other lipases *via* the formation of a complex with related protein ANGPTL8 [49]. Genetic studies have shown that carriers of loss-of-function variants in ANGPTL3 have lower plasma LDL and triglyceride levels and are at reduced risk of ASCVD [49]. Clinical studies in patients with different forms of dyslipidemia have demonstrated that inactivation of ANGPTL3 using monoclonal antibodies (evinacumab) or antisense oligonucleotides markedly lowers plasma LDL and TG levels [50]. Our study adds to the confirmation of ANGPTL3 biology specifically in the context of carotid atherosclerosis, while recent publications have extended its genetic importance also to the broader cardio-reno-metabolic context [51]. This together suggests that the beneficial effects of clinical ANGPTL3 inhibition should be evaluated broadly across the different CVD pathologies. Even more so, as we could not show any effect of currently available medications on plasma ANGPTL3 levels.

Bilirubin is the ubiquitous end-product of heme catabolism, the latter being necessary for clearance of waste products from turnover of red blood cells [11, 52] and closely associated with IPH, a widely recognized feature of atherosclerotic plaque instability [53]. We have previously demonstrated enrichment of heme catabolism in carotid plaques from symptomatic patients and shown that Biliverdin Reductase B (BLVRB), an enzyme in this pathway, is a novel biomarker of IPH associated with both the presence of IPH on carotid MRI and with ischemic stroke in patients with symptomatic carotid atherosclerosis [11, 54]. Here, BLVRA and BLVRB, as well as Bilirubin metabolites, were strongly dysregulated in various omics datasets, however in opposite trends. Both enzymes were enriched in plaques from S patients, with macrophages as the likely source, and were coupled with IPH on preoperative CTA scans. On the other hand, Bilirubin metabolites were all repressed in both local and peripheral plasma, in S vs. AS patients, and overall positively associated with Hb and erythrocyte parameters. Despite the yet unclear mechanistic relationships with CVD, the importance of this pathway has been previously suggested also from Mendelian randomization studies in the UK biobank, where genetically determined plasma Bilirubin levels were negatively associated with the risk of CVD [55]. Together, our findings suggest a complex relationship between the levels of Bilirubin generating enzymes in plaques and Bilirubin metabolites in plasma, which originate mostly from liver. This relationship could reflect IPH and unstable atherosclerosis [54], especially as Bilirubin metabolites in peripheral plasma also associated with MIs and all cause death.

Sphingomyelin metabolites were strongly enriched in both peripheral and local plasma in S patients, where they correlated positively with various lipid parameters, but also seemed to be affected by anti-diabetic medication. However, it may be that the role of Sphingomyelins is protective in this context, as their lower plasma levels systematically associated with more MACCEs and IS. Sphingomyelins are one of the classes of sphingolipids that have been described as elevated in S vs. AS patients [56, 57]. The key enzyme involved in sphingomyelin hydrolysis is acid sphingomyelinase (ASM), coded by the gene *SMPD1* [58], which when deficient, causes Neimann-Pick disease, characterized by toxic Sphingomyelin and lipid accumulation in various organs, linked to an atherogenic lipid profile and increased risk for atherosclerosis [59, 60]. This clinical observation together with the strong association between sphingolipids and CVD risk, has motivated the development of a sphingolipid-inclusive CAD score, which has been suggested to be superior to conventional clinical biomarkers [61]. Our results support this by confirming the association of Sphingomyelins with atherosclerosis and extending their prognostic value in this context.

Moreover, genetic evidence has been observed in our study for targets such as IL6 and F11 in association with CVD. Circulating levels of IL6 have been widely associated with different CVDs and outcomes, i.e. with the risk of atrial fibrillation. But also, IL6 signaling has been associated with the risk of stroke independently from atrial fibrillation [62], therefore it is not surprising that IL6 came up as one of the top candidates in our carotid cohort stratified for cerebrovascular events. Previously, genetic variants in F11 have been associated with venous thromboembolism on GWAS level [63], but recently have also been suggested as a prognostic factor in ischemic stroke of arterial origin [64]. Although historically viewed as distinct disorders with respect to etiology, pathophysiology and treatment, evidence is emerging that thromboembolic events in veins and arteries share some genetic and mechanistic features. Possibly, F11 could be at the center-point of these commonalities, as our and other recently published studies suggest also its genetic linkage with CVD events [65].

Finally, the inclusion of metabolomics data contributed towards novel discoveries in the network analyses of our multi-omics integrations, where entirely novel molecular interactomes emerged. For example, in peripheral circulation we found a direct and previously never explored association of FABP4 with various Sphingomyelins and Bilirubin, while IL6 showed links with Bilirubin degradation products. In the local disease site, there was an association of F11 and ANGPTL3 with various Sphingomyelins and cholesterol. At the intersection of plaque and peripheral disease site, a novel network appeared where several complement factors (C1S, C1R, CFB) associated with various fatty acids. Here again, IL6 interactome was linked with Bilirubin degradation products *via* complement factors, while Sphingomyelins and fatty acids formed separate interactomes. These novel networks and previously unexplored interactomes are mechanistically interesting for further studies because they could harbor significant potential for identifying biomarker-drug combinations to monitor unstable clinical atherosclerosis phenotypes.

Some limitations of our work should be mentioned. The choice of software and parameters for multi-omics integration could have affected the results, although DIABLO, which is a supervised approach, has been widely benchmarked for these types of analyses. In multi-omics integration, unsupervised methods are also often used, however, given the rich nature of the omics and clinical data available in BiKE, such choice could have led to even more complex information. Hence, we opted for a reductionist approach with DIABLO, which has been used for similar biobank studies earlier [66].

Another general challenge in integration across multiple platforms is the issue of data missingness, since technical limitations result in different omics datasets not being able to identify the corresponding analytes across all layers, essentially confounding the analysis towards the smallest, most restrictive omics dataset. Rationalizing this discordance among different omics data, whether it may be attributable to sample size, patient subphenotypes, technical variability, or differential regulation of gene vs. protein vs. metabolite networks, is key to the analysis. Interpreting the directionality of relationships within multi-omic data is another important challenge, where our results will require experimental validations. However, it is worth noting that some of these limitations are mitigated by the large size of BiKE datasets, as well as the confirmation of many targets already in clinical trials.

Even though BiKE single- and multi-omics analysis, clinical associations and survival analysis from this cohort, in combination with OpenTarget platform genetic linkage, jointly provided a systematic triaging for prioritized CVD targets, our cohort size is still limited to infer strong causal claims, due to the lack of its own genetic data layer.

Finally, the BiKE biobank exclusively contains end-stage atherosclerosis patients, with a relatively narrow age-span, which constricts our findings and limits extrapolation of conclusions into processes relevant for earlier stages of atheroprogression. Although BiKE cohort is large and well-phenotyped, it is also rather homogeneous in its composition and dominated by Caucasian descent. Therefore, results from this study should be validated in larger atherosclerosis cohorts with diverse ethnic backgrounds, where stringent corrections for multiple comorbidities and various confounding factors can be performed.

## Conclusions

In summary, this study generated some of the largest, state-of-the-art omics datasets in the field and performed first-of-a-kind multi-omics integration in human carotid atherosclerosis. Our results validate numerous previously reported pathways enriched in atherosclerotic plaques and plasma, but also reveal novel targets and pathways from the integration between local vascular disease site and peripheral circulation. Pending successful independent experimental validations, the targets presented here could have paradigm-shifting implications, especially for enabling tailored therapies and precision medicine in the fight against carotid atherosclerosis, including transfer of generated knowledge to coronary and lower limb territories (Fig. 1). As statins remain a cornerstone of treatment for all these patients, new generation of therapeutic modalities complementing or enhancing statin effectiveness and companion biomarkers, could revolutionize CVD management. By applying multi-omics to decipher the intricate systems architecture of atherosclerosis, our study provides a platform for forging novel biomarker-drug combinations that can reflect the state of plaque composition or vulnerability, and herald a new era of personalized cardiovascular care, ensuring better outcomes for patients worldwide.

## Supplementary Information

Additional file 1. Containing Figures S1-10.

Additional file 2. Containing Tables S1-11 in searchable Excel format.

## Data Availability

All data and results generated or analyzed during this study are included in this article and/or the Additional Files, which includes fully searchable Excel Tables. Created codes are publicly available at GitHub [67] . Previously, BiKE transcriptomic datasets have been deposited at NCBI Gene Expression Omnibus and made publicly available under the accession numbers GSE21545 and GSE125771 [68, 69] . BiKE plasma proteomic and metabolomic datasets have been uploaded to the public Mendeley Data database with the following link: [https://data.mendeley.com/datasets/2jzd88krks/1]. Due to the fact that individuals in the BiKE biobank are pseudonymized and not anonymised, the individual human data reported in this study cannot be deposited or shared because of the GDPR, patient consent, confidentiality agreements and ethics laws that regulate the privacy of individuals that participated in the BiKE study and prohibit publication of any personal information. All data reported in this paper on group level, summary statistics, as well as resources and reagents, may be shared upon a reasonable request to the Corresponding Author. Collaboration requests around specific targets of interest may also be directed to the Corresponding Author, who will mediate communication with the BiKE Steering Board and final decision on whether the collaboration is approved or declined due to COI, within a timeframe of several weeks-to-months.
