# Supplementary material for: Multi-omics data integration from patients with carotid stenosis illuminates key molecular signatures of atherosclerotic instability
Source: Genome Med. 2026 Feb 6;18:25. doi: 10.1186/s13073-026-01601-5 (PMC12927228; doi:10.1186/s13073-026-01601-5)
Supplement: Supplementary file 1 — Additional file 1. Containing Figures S1-10. [file 13073_2026_1601_MOESM1_ESM.docx]

**ADDITIONAL FILE I**

**Multi-omics data integration from patients with carotid stenosis illuminates key molecular signatures of atherosclerotic instability**

Vivek Das^1*^, Sampath Narayanan^2*^, Xiang Zhang^2*^, Otto Bergman^2^, Djordje Djordjevic^1^, Malin Kronqvist^2^, Melody Chemaly^2^, Glykeria Karadimou^2^, Sofija Sundman^2^, Inika Prasad^2^, Andrew J. Buckler^2^, Karin Conde Knape^1^, Natasha Barascuk Michaelsen^1^, Ulf Hedin^2^

and Ljubica Matic^2#^

^1^Novo Nordisk A/S Måløv, Denmark; ^2^Vascular Surgery, Department of Molecular Medicine and Surgery, Karolinska Institutet, Sweden

* shared first authors, ^#^ corresponding author

**Figure S1**

**Figure S1:** An illustration of all patient samples collected from carotid endarterectomy (CEA) surgery, various omics datasets and clinical/epidemiologic data used in the study. Numbers of individual patient samples (ptns) profiled with each technology are indicated under the images. PBMCs-peripheral blood monocytes.

**Figure S2**

**Figure S2:** A summary of different samples and analytical features from each layer of single-omics analyses. Abbreviations: PBMC-peripheral blood monocytes, EP-peripheral, STP-local, QC-quality control, Lpd-lipids, AmA-amino acids, Xnb-xenobiotics, Ncl-nucleotides, CaV-cofactors and vitamins, Ppt-peptides, PCM-partially characterized molecules, Crb-carbohydrates, Enr-energy, QC-quality control.

**Figure S3**

**Figure S3: Transcriptomic data analyses comparing S vs. AS patients. A)** Gene set enrichment analyses using Hallmark database, based on significantly differentially regulated genes from each comparison PBMCs (local and peripheral) or plaques**.** PBMCs-peripheral blood monocytes, AS-asymptomatic, S-symptomatic

**Figure S4**

**Figure S4: Plasma proteomic data analyses comparing S vs. AS patients. A)** Venn diagram showing overlapping proteins from comparisons of S vs. AS patients’ peripheral (EP) or local (STP) plasma. **B)** Functional enrichment analysis of proteins in local and peripheral plasma comparisons. Size of the node illustrates the number of proteins in each pathway. **C)** Evidence-based networks constructed from peripheral and local plasma proteomes comparing S vs. AS patients, with key drivers indicated (red-increased, green-decreased in respective sites). AS-asymptomatic, S-symptomatic, FC-fold change.

**Figure S5**

**Figure S5: Plasma metabolomic data analyses comparing S vs. AS patients. A)** Venn diagram shows overlapping metabolites from plasma analysis comparing S vs. AS patients, both local (STP) and peripheral (EP) blood. **B)** Enrichment analysis of metabolites in local and peripheral blood from S vs. AS patients. Size of the node illustrates the number of metabolites in each pathway. Dashed line corresponds to p<0.05. AS-asymptomatic, S-symptomatic, FC-fold change.

**Figure S6**

**Figure S6:** Overlaps among the various omics datasets with numbers of intersection features (molecules/analytes) indicated in the blue bar chart to the left, while numbers of patients in intersections are indicated above the black bar chart to the right

**Figure S7**

**Figure S7:** A summary of different samples and analytical features from each layer of multi-omics analyses. The numbers of all significant features (molecules/analytes) that were found in each omics layer from the indicated Combination for Symptomatic vs. Asymptomatic stratification are indicated in the table, as well as the numbers of patient samples. PBMCs-peripheral blood monocytes.

**Figure S8**

**Figure S8:** Heatmap representation of correlations between transcript, protein or metabolite levels for top targets of interest from omics data and various clinical biochemistry parameters from patients. Correlations were done using Spearman rho method (scale to the right), with correction for multiple comparisons (p-value<0.05 after adjustment indicated with asterisk in the plots). Units for the clinical parameters are as follows: S_Creatinine (serum creatinine, umol/L), CRP (C reactive protein, mg/L), LPK (10^6^/L), Hb (hemoglobin, %), erythrocytes (10^12^/L), EVF (erythrocyte volume fraction, %), MCV (mean corpuscular volume, fL), MCH (mean corpuscular hemoglobin, pg), MCHC (mean corpuscular hemoglobin concentration, pg), HbA1c (acetylated hemoglobin, mmol/mol), S_Cholesterol (mmol/L), LDL (low density lipoprotein, mmol/L), HDL (high density lipoprotein, mmol/L), TG (triglycerides, mmol/L), Fibrinogen (g/L), eGFR (mL/min/1.73m2), Bilirubin (umol/L), Proinsulin (pmol/L), IL6 (ng/L), Adiponectin (mg/L). PBMCs-peripheral blood monocytes.

**Figure S9**

**Figure S9:** Forest plot representation of associations between transcript, protein or metabolite levels for top targets of interest from omics data and various medications in the BiKE database. Only selected plots are shown with significant or near-significant results. Confidence intervals (CI) indicated in the x-axis, p-value after adjustment for age and sex indicated in the plots. Medications tested were: Lipid-decreasers (No: 399 patients, Yes: 673), anti-diabetics (No: 363 patients, Yes: 219), anti-hypertensives, and anti-thrombotics (No: 166, Yes: 857). PBMCs-peripheral blood monocytes.

**Figure S10**

**Figure S10: Cumulative Kaplan-Meier outcome estimates after carotid surgery.** Plots illustrating survival of BiKE patients during the 15 years follow-up period after surgery, based on top *vs.* bottom quartile of relevant analyte levels. Both patients that were symptomatic and asymptomatic at surgery were included in the follow-up analysis. Each mark along the lines indicates an event, numbers at risk indicated in tables under the plots. Top raw shows plots for plaque ICOSLG transcript levels. Middle raw shows plots for BLVRB plaque transcript levels, and bottom raw shows FABP4 plaque transcript expression. MACCEs-major adverse cardio- and cerebro-vascular events; MI-myocardial infarction.
